# Taxonomic characterizations of the genus *Commicarpus* Standl. (Nyctaginaceae) in Saudi Arabia

**DOI:** 10.1371/journal.pone.0350149

**Published:** 2026-05-28

**Authors:** Maad S. Ytemi, Hameed Alsamadany, Faraj Abdullah Al-Ghamdi, Abadi M. Mashlawi, A. El-Shabasy

**Affiliations:** 1 Department of Biological Sciences, Faculty of Science, King Abdulaziz University, Jeddah, Saudi Arabia; 2 Department of Biology, College of Science, Jazan University, Jazan, Saudi Arabia; Anhui University of Chinese Medicine, CHINA

## Abstract

The genus *Commicarpus* Standl. is a member of the family Nyctaginaceae. The genus includes about 30–35 species distributed across tropical and subtropical regions worldwide, including Saudi Arabia. Five species of *Commicarpus* are found through, the field survey, which are primarily concentrated in the western and southwestern regions of Saudi Arabia. The collected species are *C. grandiflorus*, *C. helenae*, *C. mistus*, *C. plumbagineus*, and *C. sinuatus*. The aim of this study is to do morphological, anatomical, and palynological analyses of these species. Morphologically, growth habit, stem texture, leaf characteristics, floral structure, and fruit morphology were evaluated, these characters are significant to distinguish *Commicarpus* species. Anatomical studies of the stems, leaves, and petioles show some important characteristics that can help to separate *Commicarpus* species, including variations in collenchyma and chlorenchyma layers, vascular bundle arrangement, and mesophyll structure. Petiole anatomy, particularly the shape and arrangement of ground tissue and vascular bundles, provides additional taxonomic markers. Also, the study of pollen grains of the species using light microscopes (LM) and scanning electron microscopes (SEM) provides significant character that can be used for species differentiation, including differences in pollen size, shape, polar and equatorial axis dimensions, tubuliferous density, pore diameter, and spinule length. Pollen grains are very large in *C. grandiflorus*, *C. plumbagineus*, and *C. sinuatus* and large in *C. helenae* and *C. mistus*; their shapes range from oblate-spheroidal in *C. grandiflorus* to prolate-spheroidal in the other species. Two keys are constructed, one utilizing morphological characteristics and the other employing anatomical features of the petioles to aid in species identification. These results contribute valuable taxonomic information for the genus *Commicarpus* in Saudi Arabia.

## Introduction

A large part of the Arabian Peninsula is made up of Saudi Arabia, a vast arid desert covering over 225,000 square kilometers. It is frequently thought of being a nation with mostly desolate terrain and little greenery [1]. With the exception of mosses and grasses, a research by Collenette (1999) [2] revealed an astounding biodiversity of 2,250 plant species. over 855 genera and 2,282 species from 131 families make up this varied flora. The northwestern and southwestern regions have the highest concentration of plant species, making up over 70% of the country’s floral diversity. According to current estimates, there are around 1,620 (71.02%) herbaceous plants, 565 (24.73%) shrubs, and 97 (4.25%) trees in Saudi Arabia [3].

The Nyctaginaceae Jussieu is a compact family comprising approximately 30 genera and 400 species [4]. It is often referred to as the Four-O’Clock family due to the characteristic of many species having flowers that bloom from late afternoon to early evening [5]. The Nyctaginaceae family is primarily found in tropical and subtropical regions of the New World [6,7], particularly the Americas [8], with some genera also extending into temperate areas such as southern Africa, Western Asia, East Asia, and Australia [4,9–13]. The family has two main centers of distribution in the Americas: tropical and subtropical South America and the Antilles, as well as the southwestern United States and northern Mexico in North America [6]. However, certain genera like *Boerhavia*, *Commicarpus*, *Pisonia*, and *Mirabilis* are found in the Old World, specifically in South Africa, with *Phaeoptilum* being restricted to Africa [4].

*Commicarpus* Standl. is a genus within the Nyctaginaceae family, alongside other genera like *Boerhavia* L., *Mirabilis* L. and *Pisonia* L. and This genus comprises approximately 30–35 species native to tropical and subtropical regions, with a significant presence in Africa and western Asia [6,14]. *Commicarpus* species are characterized by unique anthocarp details, flower morphology, and growth preferences in calcium-rich soil with heavy metal components [4,15]. The genus *Commicarpus* standl. is widely distributed across tropical and subtropical regions, with a notable presence in Africa, Western Asia, East Asia, Australia, and the Americas 2, 6, 8, 9,10, 11, 12, 13, 14, 16]. Within the Kingdom of Saudi Arabia, this genus has estimates ranging from 3 to 7 species [2,9,11,13,16]. These species are primarily concentrated in the western and southwestern regions of Saudi Arabia.

Struwig et al. (2011) [17,18] studied the anatomy of *Boerhavia* and *Commicarpus* species from the Nyctaginaceae family in southern Africa, investigating how these plants have adapted anatomically to live in Namibia’s arid environments. A systematic revision of *Commicarpus* species in South Africa was carried out by Struwig & Siebert (2013) [15], most likely comprising the identification, categorization, and description of species in this genus. Pakravan et al. (2023) [19] used a variety of methods, including morphological investigations, to conduct a systematic investigation of Nyctaginaceae species in Iran. In order to identify species within the Nyctaginaceae family in Iran, they focused on finding important traits. The shapes, sizes, and arrangements of leaves, stems, flowers, and other exterior aspects were among the general physical characteristics and plant structures that they looked at. Additionally, the anatomical study investigated differences in vascular bundles, petioles, phloem, xylem, stomatal types, and epidermal cells in the internal structures and tissues of Iranian Nyctaginaceae plants. In order to classify plants taxonomically and comprehend their ecological responsibilities, this kind of anatomical investigation offers insights into the physiological processes and adaptations of the plants. Struwig et al. (2013) [20] examined the pollen morphology of southern African species of *Boerhavia* and *Commicarpus*, probably in an effort to comprehend the properties of pollen grains and their possible importance in plant identification and evolutionary research. The distinctive characteristics and morphology of the pollen grains of the Nyctaginaceae species in Iran were described by Pakravan et al. (2023) [19]. This palynological approach can offer further evidence to support the taxonomic location and connections within the Nyctaginaceae family since pollen grains have unique sizes, shapes, and surface patterns that can be utilized to distinguish between species.

Despite slight variations in habit and leaf shape, Meikle, (1978) [21] noted that all *Commicarpus* species are superficially similar in growth form and foliage, making it challenging to distinguish them in the field. This raises the question of how to reliably differentiate between *Commicarpus* species based on observable characteristics. There is inconsistency in the species of the genus *Commicarpus* recorded in the references of the Flora of Saudi Arabia and Arabian Peninsula. This discrepancy highlights the need for clarification and standardization of the species composition within the genus in the region.

The aim of this study is to evaluate the taxonomic value of *Commicarpus* species; *C.* *grandiflorus* (A.Rich.) Standl., *C. helenae* (Schult.) Meikle, *C. mistus* Thulin, *C. plumbagineus* (Cav.) Standl., and *C. sinuatus* Meikle within Saudi Arabia based on morphological, anatomical, and palynological characterizations.

## Materials and methods

### Specimen collection for morphological studies

**Fig 1:**
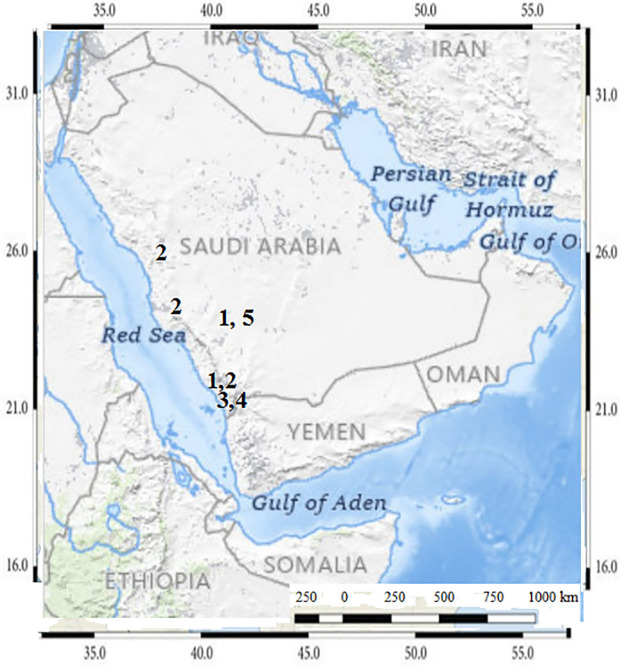
Map of studied area with localities of *Commicarpus* species; (1) *C. grandiflorus*; (2) *C. helenae;* (3) *C. mistus;* (4) *C. plumbagineus;* (5) *C. sinuatus.* The map is designed according to USGS National Map Viewer (public domain): http://viewer.nationalmap.gov/viewer/. The Gateway to Astronaut Photography of Earth (public domain): http://eol.jsc.nasa.gov/sseop/clickmap/.

**Fig 2:**
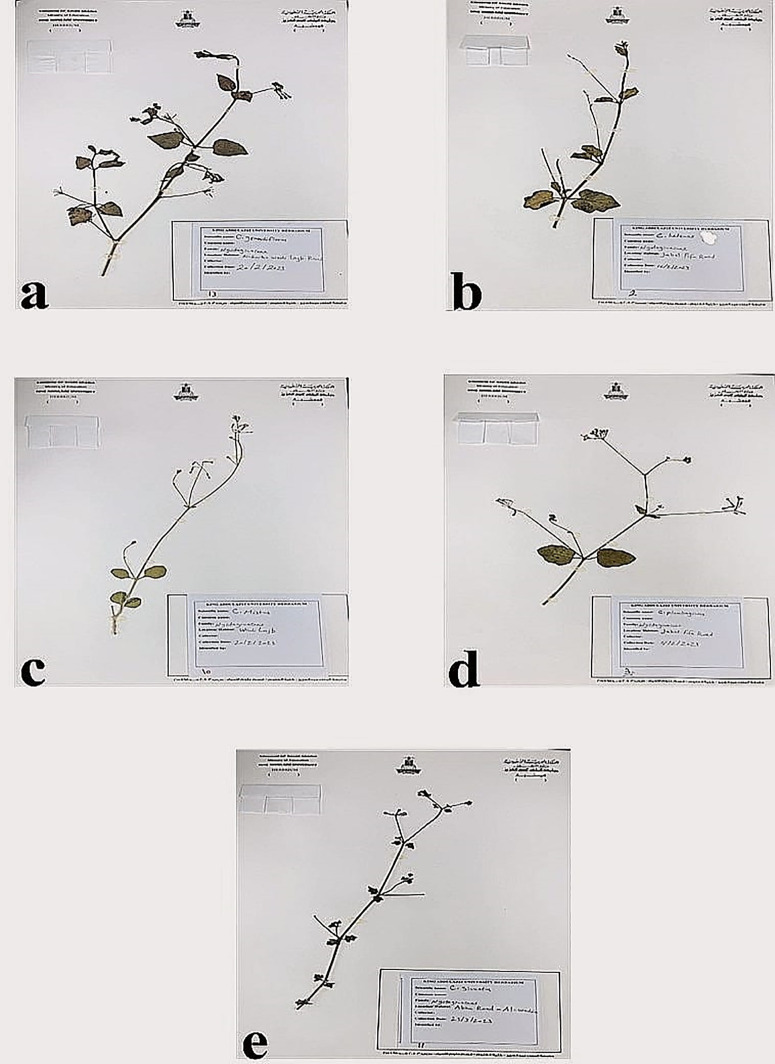
Herbarium specimens of *Commicarpus* species collected from various locations within Saudi Arabia, identified by the Herbarium of King Abdulaziz University, and subsequently stored there. Herbarium specimens of the studied species; **(a)** *C. grandiflorus*; **(b)** *C. helenae;* **(c)** *C. mistus;* **(d)** *C. plumbagineus;* **(e)** *C. sinuatus.*

The studied area is extended north from Al-Madinah Al-Munawwarah and Makkah Al-Mukarramah, then downwards into the southwestern regions at Asir and Jazan. In the present study, samples of *Commicarpus* species were collected from various regions of Saudi Arabia (Table 1 and Fig 1), as referenced in the Flora of Saudi Arabia and Arabian Peninsula, documented by [2,9,11,13,16]. These sources provide evidence that these species exhibit a predominant distribution in the western and southwestern regions of Saudi Arabia. The collection specimens through 2023 were carefully preserved as herbarium specimens [22] and stored at the King Abdul-Aziz University herbarium (Fig 2). To enhance reproducibility, all specimens were geo-referenced using GPS coordinates, and detailed collection metadata—including habitat type, altitude, and sampling date—were systematically recorded following standardized biodiversity sampling practices.

**Table 1:** Localities of the five studied *Commicarpus* species.

No.	species	Location	Date of collection	Coordinates	Elevation (m)*	Collector	Voucher specimens	Identifier
**1**	***C. grandiflorus***	**Asir region:**						King Abdulaziz Herbarium (Dr. Faraj Al Ghamdi)
		Al-Wadieen.	23 - 3- 2023	18.118855,42.859390	2109		KAU-12	
		**JAZAN:**						
		Al-Jabal Al-Aswad – Wadi Lajb Road.	20 −2 −2023	17.580055,42.898361	1384	M. Ytemi	KAU-13	
		Jabal Al-Hashr Road.	20 - 2 −2023	17.452274,43.054977	2048		KAU-7	
		Jabal Fayfa Road.	8 - 3 −2023	17.248472,43.094566	1378		KAU-17	
		Jabal Al-Abadil Road.	14 - 3 −2023	17.015892,43.145065	1065		KAU-21	
**2**	***C. helenae***	**Al-Madinah Al-Munawwarah:**						King Abdulaziz Herbarium (Dr Faraj Al Ghamdi).
		The Road to Al-Faqrah village.	23 - 5 −2023	24.322893,39.048443	1013		KAU-24	
		**Makkah Al-Mukarramah:**				M. Ytemi		
		Al-Zaimah.	27 - 2 −2023	21.617161,40.106277	562		KAU-14	
		**JAZAN:**						
		Jabal Fayfa Road.	16 - 2 −2023	17.239475,43.061478	694		KAU-2	
		Abk Village – Jabal Sala Road.	17 - 2 −2023	17.029114,43.140701	669		KAU-5	
**3**	***C. mistus***	**JAZAN:**						King Abdulaziz Herbarium (Dr. Faraj Al Ghamdi)
		Al-Jabal Al-Aswad – Wadi Lajb Road.	20 - 2 −2023	17.577814,42.916378	1312	M. Ytemi	KAU-10	
**4**	***C. plumbagineus***	**JAZAN:**						King Abdulaziz Herbarium (Dr. Faraj Al Ghamdi)
		Khatwat Al-Ain – Wadi Lajb Road.	20 - 2 −2023	17.597113,42.930325	1321		KAU-8	
		Jabal Al-Hashr Road.	20 - 2 −2023	17.452274,43.054977	2046	M. Ytemi	KAU-6	
		Jabal Fayfa Road.	16 - 2 −2023	17.238794,43.064541	809		KAU-3	
		Abk Village – Jabal Sala Road.	17 - 2 −2023	17.029114,43.140701	669		KAU-4	
		Abk Village – Jabal Al-Abadil Road.	14 - 3 −2023	17.018459,43.142746	1089		KAU-20	
**5**	***C. sinuatus***	**Asir region:**						King Abdulaziz Herbarium (Dr. Faraj Al Ghamdi)
		Al-Wadieen.	23–3 −2023	18.118855,42.859390	2109	M. Ytemi	KAU-11	

Subsequently, measurements and observations of 100 replicas for each species were conducted to record various vegetative characteristics, including leaf features such as length, petiole length, arrangement, lamina shape, length, width, apex, base shape and margin structure. Also, these reproduction *Commicarpus* species were observed: inflorescence type, length, hair characteristics and surface texture, as well as flower attributes including length, pedicel length, petaloid region length, coriaceous length, perigonium shape and color, stamens number and length, ovary and stigma shape, number of ovules, and the presence or absence of bracts. Additionally, fruit shape, length, and indumentum were documented. All measurements were performed using calibrated digital calipers, and replicate measurements were averaged to minimize observational error and improve data reliability.

### Anatomical preparations

Fresh samples of stem, leaf, and petiole were collected from various locations in Saudi Arabia during 2023. Utilizing preparations of [23–25], the specimens were collected and prepared for cross-sectional permanent slides. The plant parts (stem, leaf, and petiole) were fixed in a fixative solution consisting of 50 ml absolute ethyl alcohol, 10 ml concentrated formalin, 5 ml glacial acetic acid, and 35 ml distilled water [23]. After appropriate fixation time, the preserved samples were cut into small pieces (0.5 cm) using a sharp scalpel and passed through a graded series of ethyl alcohol (30%, 50%, 70%, 80%, 90%, 95%, and absolute alcohol) for one hour at each concentration [24]. The samples were passed through a series of ethyl alcohol-xylene mixtures in the following volume ratios: 3:1, 1:1, and 1:3. Then, they were placed in pure xylene for two hours. Samples were transferred from xylene (after clearing) to a 1:1 mixture of xylene and liquid paraffin and left in the oven at 60°C overnight to replace the evaporated xylene with liquid paraffin. The liquid paraffin-xylene mixture was poured out and replaced with pure liquid paraffin, and the samples were left in the oven for two hours. The samples were then immersed in wax overnight to ensure proper impregnation. For embedding and trimming, the plant parts were positioned inside aluminum corner molds covering with amount of liquid paraffin, then labeled and left cool at lab temperature. The wax cubes containing the plant parts were then placed in the freezer until they were fully hardened and ready for trimming [26]. The cubes (containing the plant parts) were trimmed using a special blade and placed on a microtome. The samples were cut into 12-micron-thick sections for stem, leaf, and petiole in the form of paraffin ribbons. These ribbons were placed in a water bath at 40°C to flatten them. The ribbons were lifted using clean glass slides precoated with a thin smear of glycerin-albumin adhesive. The slides were tilted slightly to remove water and then placed on a hot plate (35–40°C) for 4–12 hours to fix the section ribbons and remove wrinkles. The slides were then ready for dewaxing and staining. Paraffin wax was removed from both inside and outside the tissue by placing the slides containing the sections in the oven to melt the wax thoroughly. The glass slides were then passed through xylene and ethyl alcohol concentrations as follows: 100% pure xylene, 3:1 xylene and ethyl alcohol for five minutes, 1:1 xylene and ethyl alcohol for five minutes, and 1:3 xylene and ethyl alcohol for five minutes. Subsequently, the slides were passed through a descending series of ethyl alcohols (100, 95, 90, 80, 70, 50, 30) for 5 minutes at each concentration. The slides were then stained with safranin (1% w/v dissolved in 50% ethanol) and fast green (0.5% w/v dissolved in 95% ethanol) stains and examined. Staining quality and section integrity were verified microscopically prior to imaging to ensure accurate tissue differentiation and to minimize preparation artifacts. The prepared slides were examined and documented using standard light microscopy techniques [27].

### Palynological studies

In the current study, pollen from fresh plant material collected in Saudi Arabia was examined using light microscopy (LM) and scanning electron microscopy (SEM) to analyze its morphology and ultrastructure. The pollen was prepared using the acetolysis method according to [28]. The acetolysis method was applied to ensure consistency. The procedure was conducted under controlled conditions to preserve exine structure while effectively removing cytoplasmic material, which is essential for accurate palynological analysis. Flowers containing mature anthers were collected, and the anthers were separated using fine forceps and a dissecting needle. The isolated anthers were transferred onto Pollen materials of each species were removed from the anthers of well-developed flower buds near to anthesis in a Petri dish and placed in a centrifuge tube with 5 mL acetic acid, then remaining for at least 24 hours before acetolysis. Centrifuge was done at 1500–1800 revolutions per minute/rpm for 5–10 min and the supernatant was decanted then 4.5 mL of acetic anhydride and 0.5 mL of sulfuric acid (9:1) were placed in the centrifuge tube. Immediately the centrifuge tubes were dipped with the acetolysis mixture into a beaker containing hot water in a laboratory water bath close to boiling from 80 to 100 ° C. Subsequently a glass rod in each tube was placed and gently mixed the contents at regular intervals. The water bath was kept boiling slowly for 1–2 min then the bath must be placed in a fume hood to avoid nasal aspiration of vapors, which are very irritating and toxic. After that centrifuge was done for five minutes at 1500–1800 rpm then the supernatant was discarded. Distilled water was added to the pollen residue to make up the volume to 10 ml. Each tube was shaken with one by one the two drops of ethyl alcohol. After centrifugation and decantation, a mixture of 5 ml of water with glycerin in equal parts was added to pollen residues. Let it remain in this solution for half an hour or until the next day. Finally, a cube of glycerin gelatin about 1 mm3 that was inserted into the bottom of the centrifuge tube to collect the pollen residue to be placed in the center of the microscope slide and gelatin melting process is performed carefully under heat to avoid boiling. The coverslip was placed in the center of the slide and sealed with paraffin and examined under a light microscope. The prepared slides were examined using an Olympus light microscope equipped with an oil immersion lens. A total of 10–20 pollen grains from each species were analyzed. Measurements included the equatorial axis diameter, the polar axis length, spinule length, and pore diameter, all recorded using a calibrated ocular micrometer. For SEM, pollen grains were released from the anther samples and fixed on stubs using double-sided adhesive tape; the samples were then coated with a 150-angstrom-thick layer of gold and examined using a Jeol JEM 5400 LV SEM. Palynological descriptions follow the terminology established by [29]. Imaging parameters and coating thickness were standardized to ensure comparability of surface ornamentation and to minimize imaging bias.

### Scoring data and statistical analysis

To establish a phenetic analysis, every morphological and anatomical characteristic of the species under study was assessed. The Pclass approach was used to build the similarity matrix and cluster analysis. The distances were calculated based on the Gower coefficient. The pairwise similarities and dissimimilarites between the operational taxonomic units (OTUs) were determined using the Nei genetic similarity index (SI) and the equation SI = 2Nij/ (Ni + Nj), where Ni and Nj represent the total number of comparative characters for each species i and j, respectively, and Nij represents the number of common characters shared between them. The phenogram was generated using a sequential agglomerative hierarchical nested clustering approach. This method, known as unweight pair group mathematical averages (UPGMA), involved combining previously studied fern species with similar characteristics through a series of successive mergers [25]. The Pearson correlation coefficient was used among morphological *vs* anatomical parameters, morphological *vs* palynological parameters finally anatomical *vs* palynological parameters in representation of simple linear regression (SRL) to estimate the degree of affinity between each characteristic parameters and determine the compatibility level that influenced on *Commicarpus* species by using methods according to [30–32]. *P* values, which were employed as significant parameters, were determined by applying [33] techniques based on the degree of freedom. All statistical analyses were conducted using standardized multivariate approaches to ensure robustness and reproducibility.

### Species synopsis

The species synopsis includes a taxonomic treatment of the genus *Commicarpus* Standl., which encompasses a description of the studied species within this genus in Saudi Arabia. It also involves the development of a detailed taxonomic key to facilitate species identification based on distinctive morphological, and anatomical characteristics. This work aims to resolve taxonomic ambiguities and enhance the understanding of *Commicarpus* diversity within Saudi Arabia.

## Results

### Morphological analysis

#### Stem morphology (Fig 3 and Table 2).

The analysis of stem morphology in *Commicarpus* species from Saudi Arabia revealed distinct growth forms and notable variations in stem characteristics across the five studied taxa.

**Table 2:** Summary of the growth forms and stem characterizations of *Commicarpus* species.

Species	Characters					
	Stem					
	Growth form			Length (m)	Hairs	Texture
	Habit	Branching pattern	Growth orientation			
***Commicarpus grandiflorus***	Perennial herbs, soft woody	Slightly branched	accumbent or ascending	(1.7 -) 1.8 (- 2.0)	Pilose-glandular hairs	Sticky
***Commicarpus helenae***	Perennial herbs, woody based	Much branched	Suberect to scrambling	(1.0 -) 1.3 (- 2.0)	Glabrous	Non-sticky
***Commicarpus mistus***	Perennial herbs, woody based	Slightly branched	Erect	(0.7 -) 0.8 (- 0.9)	Puberulent	Non-sticky
***Commicarpus plumbagineus***	Perennial herbs, soft woody	Slightly branched	Scandent or trailing	(1.2 -) 1.9 (- 3.0)	Glabrous	Non-sticky
***Commicarpus sinuatus***	Perennial herbs, woody based	Much branched	Tangled	(1.8 -) 2.4 (- 3.0)	Glabrous	Non-sticky

**Fig 3:**
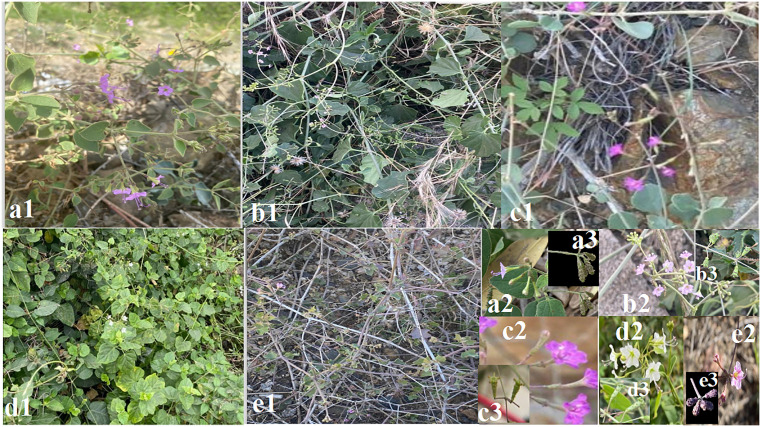
Saudi Arabian *Commicarpus* species 1: Growth forms, 2: Flowers, 3: Fruits (a) *C. grandiflorus*; (b) *C. helenae*; (c) *C. mistus*; (d) *C. plumbagineus*; (e) *C. sinuatus.*

All examined species exhibit a perennial herbaceous habit with variations in woody development. *Commicarpus grandiflorus* and *C. plumbagineus* are characterized as soft woody, whereas *C. helenae*, *C. mistus* and *C. sinuatus* display a woody-based structure. Branching patterns range from slightly branched to much branched forms. *Commicarpus grandiflorus*, *C. mistus* and *C. plumbagineus* exhibit a slightly branched structure, contributing to their more linear growth habits. In contrast, *C. helenae* and *C. sinuatus* are highly branched, leading to a more complex and expansive growth form. The orientation of growth further differentiates the species: *C. grandiflorus* displays an accumbent or ascending habit, *C. helenae* grows suberect to scrambling, *C. mistus* maintains an erect posture, *C. plumbagineus* exhibits scandent or trailing behavior, and *C. sinuatus* is characterized by a tangled growth pattern.

Significant variation in stem length was observed among the species. *C. sinuatus* has the longest stems, measuring (1.8–) 2.4 (–3.0) m, followed by *C. plumbagineus* (1.2–) 1.9 (–3.0) m, *C. grandiflorus* (1.7–) 1.8 (–2.0) m, and *C. helenae* (1.0–) 1.3 (–2.0) m. In contrast, *C. mistus* has the shortest stems, ranging from (0.7–) 0.8 (–0.9) m.

The species also exhibit considerable differences in stem surface features. *C. grandiflorus* is unique in possessing pilose-glandular hairs and a sticky surface texture. *C. mistus* is characterized by puberulent hairs. In contrast, *C. helenae*, *C. plumbagineus* and *C. sinuatus* have glabrous stems. Notably, all species, except *C. grandiflorus*, have a non-sticky surface texture.

#### Leaf morphology (Fig 4 and Table 3).

The leaf characteristics of *Commicarpus* species exhibit considerable variation in several aspects, including leaf and petiole length, leaf arrangement, lamina shape, lamina dimensions, apex and base shape, and margin structure.

**Table 3:** A summary of the leaf characteristics of *Commicarpus* species in Saudi Arabia.

Species	Characters								
	Stem								
	Length (mm)	Petiole length (mm)	Lamina						
			Texture	Shape	Length (mm)	Width (mm)	Apex	Base	Margin
***Commicarpus grandiflorus***	(23.0 -) 33.3 (-71.0)	(3.0 -) 8.1 (- 12.0)	Non-fleshy	Ovate-triangular	(20.0 -) 25.6 (- 59.0)	(10.0 -) 17.9 (- 26.0)	Acute or obtuse	Truncate to subcordate	Entire
***Commicarpus helenae***	(10.0 -) 24.1 (- 43.0)	(3.0 -) 5.9 (- 8.0)	Fleshy	Broadly ovate	(7.0 -) 17.8 (- 35.0)	(3.0 -) 14.8 (- 28.0)	Acute or obtuse	Cordate	Entire or sinuately lobed
***Commicarpus mistus***	(7.0 -) 13.7 (- 27.0)	(2.0 -) 3.5 (- 7.0)	Fleshy	Ovate or sub-orbicular	(5.0 -) 12.2 (- 20.0)	(4.0 -) 7.3 (- 17.0)	Obtuse or rounded	Truncate to broadly cuneate	Entire or obscurely sinuate
***Commicarpus plumbagineus***	(27.0 -) 38.5 (- 86.0)	(4.0 -) 11.6 (- 20.0)	Non-fleshy	Broadly ovate	(23.0 -) 29.5 (- 66.0)	(15.0 -) 24.4 (- 47.0)	Acute or obtuse	Truncate to broadly cuneate	Entire to irregularly sinuate
***Commicarpus sinuatus***	(6.0 -) 10.5 (- 13.0)	(1.0 -) 2.0 (- 3.0)	Fleshy	Sinuate or lobed	(5.0 -) 8.7 (- 10.0)	(4.0 -) 7.7 (- 10.0)	Obtuse	Truncate to subcordate	Sinuately lobed

**Fig 4:**
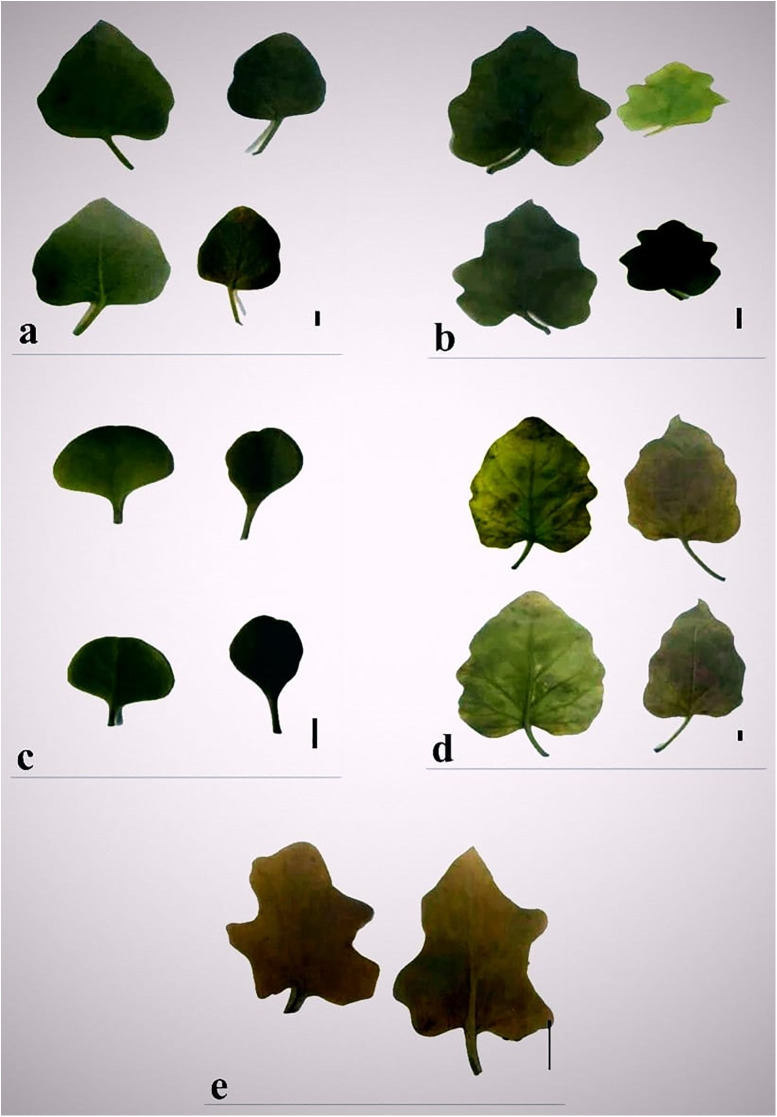
Leaf shapes of *Commicarpus* species (a) *C. grandiflorus*; (b) *C. helenae;* (c) *C. mistus*; (d) *C. plumbagineus*; (e) *C. sinuatus.* Scale bars 10 mm.

Among the studied species, *C. plumbagineus* has the largest leaves, with lengths ranging from (27–) 38.5 (–86) mm, whereas *C. sinuatus* and *C. mistus* possess the smallest leaves, measuring (6.0–) 10.5 (–13) mm and (7.0–) 13.7 (–27) mm, respectively. Petiole lengths also vary significantly, with *C. plumbagineus* having the longest petioles (4.0–) 11.6 (–20) mm, while *C. sinuatus* has the shortest (1.0–) 2.0 (–3.0) mm. Despite differences in size and shape, all species exhibit an opposite leaf arrangement. Two species, *C. grandiflorus* and *C. plumbagineus*, exhibit non-fleshy leaves, while the remaining species (*C. helenae*, *C. mistus*, and *C. sinuatus*) possess fleshy leaves, indicating varying degrees of succulence that may correlate with drought tolerance.

Lamina shape displays considerable diversity among species. *C. grandiflorus* has ovate-triangular leaves, while C. *helenae* and *C. plumbagineus* feature broadly ovate forms. *C. mistus* exhibits ovate to sub-orbicular leaves, whereas *C. sinuatus* is characterized by sinuate or lobed leaves, a feature that distinguishes it from the other species.

Significant variation is also observed in lamina dimensions. *C. plumbagineus* has the longest lamina, measuring (23–) 29.5 (–66) mm, and the widest lamina, ranging from (15–) 24.4 (–47) mm. In contrast, *C. sinuatus* has the shortest lamina at (5.0–) 8.7 (–10) mm and the narrowest lamina at (4.0–) 7.7 (–10) mm.

The lamina apex varies between acute, obtuse, and rounded forms. *C. grandiflorus*, *C. helenae*, and *C. plumbagineus* exhibit acute to obtuse apices, while *C. mistus* and *C. sinuatus* are characterized by obtuse or rounded apices.

The lamina base shows notable diversity: *C. helenae* is distinct with a cordate base, while the other species display truncate to subcordate (*C. grandiflorus*, *C. sinuatus*) or truncate to broadly cuneate (*C. mistus*, *C. plumbagineus*) bases.

Lamina margin morphology serves as a key diagnostic character among the studied species. *C. grandiflorus* possesses entire margins, while *C. helenae* and *C. mistus* display either entire or sinuately lobed/obscurely sinuated margins. *C. plumbagineus* exhibits entire to irregularly sinuated margins, and *C. sinuatus* has distinctly sinuately lobed margins, contributing to its unique identification.

#### Inflorescence morphology (Figs 5 and 6 and Table 4).

The inflorescence characteristics of *Commicarpus* species vary significantly in type, surface texture, hair characteristics, and length. *C. grandiflorus*, *C. mistus*, and *C. sinuatus* produce umbels, whereas *C. helenae* and *C. plumbagineus* primarily exhibit umbels arranged in groups.

**Table 4:** A summary of the Inflorescence, fruit, and bracts characteristics of *Commicarpus* species in Saudi Arabia.

Species	Characters							
	Inflorescence				Fruit			Bracts
	Type	Hairs	Surface texture	Length (mm)	Shape	Length (mm)	Indumentum	
***Commicarpus grandiflorus***	Umbels	Glandular- hairy	Sticky	(30.0 -) 34.8 (- 41.0)	Clavate	(5.0 -) 6.3 (- 7.0)	Fruit covered with numerous sessile glands and finely viscid pubescent	Absent
***Commicarpus helenae***	In whorls	Glabrous	Non-sticky	(35.0 -) 51.2 (- 70.0)	Clavate	(4.0 -) 4.8 (- 6.0)	Fruit covered with long-stalked glands at the tip, inconspicuous glandular below.	Present
***Commicarpus mistus***	Umbels	Puberulent	Non-sticky	(21.0 -) 24.2 (- 30.0)	Clavate	(6.0 -) 7.4 (- 8.0)	Fruit covered with puberulent hairs and prominent long-stalked glands at the tip, inconspicuous glandular below.	Absent
***Commicarpus plumbagineus***	Mostly in Whorls	Glabrous	Non-sticky	(32.0 -) 42.5 (- 60.0)	Fusiform	(7.0 -) 8.0 (- 9.0)	Fruit covered with prominent sessile glands, mostly at the apex	Absent
***Commicarpus sinuatus***	Umbels	Glabrous	Non-sticky	(17.0 -) 22.6 (- 30.0)	Clavate	(5.0 -) 6.7 (- 8.0)	Fruit covered with sessile glands, scattered all over	Absent

**Fig 5:**
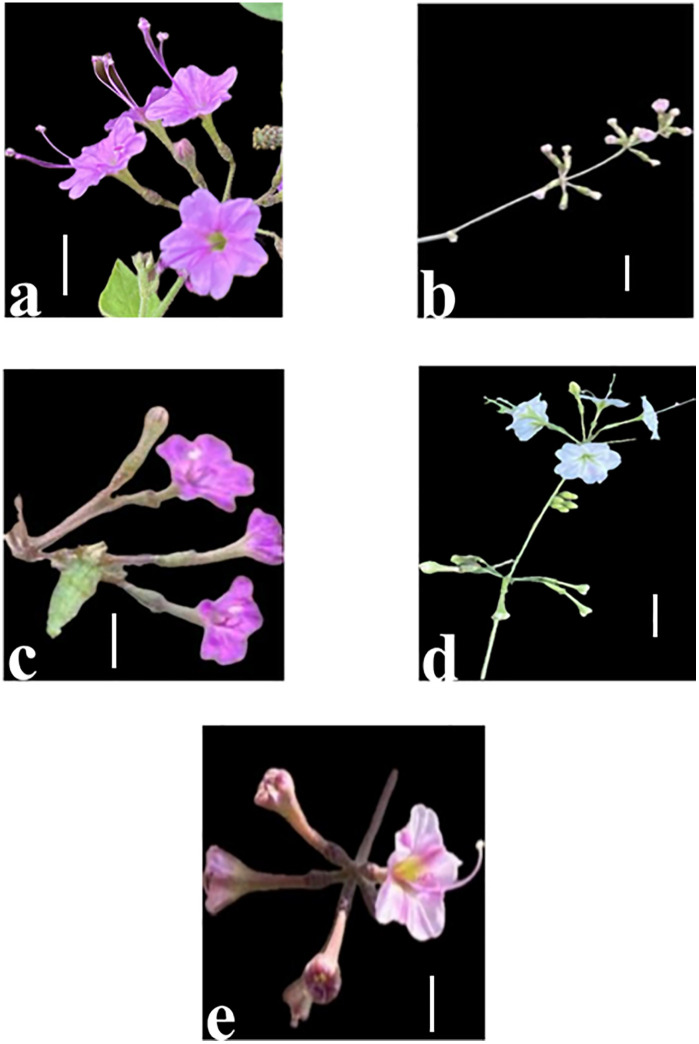
Inflorescences of *Commicarpus* species: (a) *C. grandiflorus*; (b) *C. helenae*; (c) *C. mistus*; (d) *C. plumbagineus*; (e) *C. sinuatus.*

**Fig 6:**
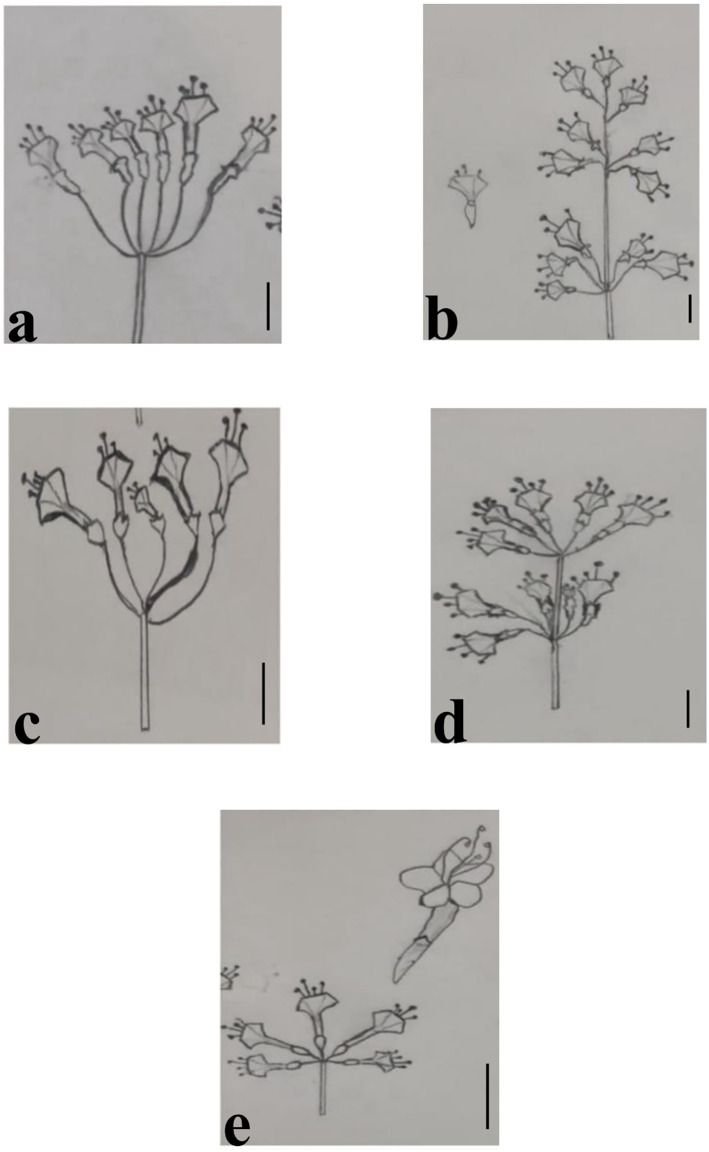
Inflorescences of *Commicarpus* species: (a) *C. grandiflorus*; (b) *C. helenae*; (c) *C. mistus*; (d) *C. plumbagineus*; (e) *C. sinuatus.* Scale bars 10 mm.

Surface texture also differs among species. The inflorescences of *C. grandiflorus* are glandular-pilose and sticky, while those of *C. mistus* are puberulent and non-sticky. In contrast, *C. helenae*, *C. plumbagineus*, and *C. sinuatus* have glabrous and non-sticky inflorescences.

Inflorescence length varies among species. *C. helenae* has the longest inflorescences, measuring (35–) 51.2 (–70) mm, whereas *C. mistus* and *C. sinuatus* possess the shortest inflorescences, measuring (21–) 24.2 (–30) mm and (17–) 22.6 (–30) mm, respectively.

#### Flower morphology (Fig 7 and Table 5).

The flower characteristics of *Commicarpus* species exhibit significant variation across multiple traits, including flower and pedicel length, petaloid region and coriaceous segment dimensions, perigonium shape and colour, stamen number and length, ovary shape, ovule count, stigma structure, and the presence of bracts.

**Table 5:** A summary of the flower characteristics of *Commicarpus* species in Saudi Arabia.

Species	Characters							
	Flower							
	Length (mm)	Pedicel length (mm)	Perianth part		Perigonium		Stamen	
			Petaloid region length (mm)	Coriaceous length (mm)	Shape	Colour	Number	Length (mm)
***Commicarpus grandiflorus***	(13.5 -) 16.6 (- 21.0)	(5.5 -) 6.2 (- 7.0)	(6.0 -) 7.5 (- 9.0)	(2.0 -) 3.3 (- 5.0)	Narrowly infundibuliform, with a distinct tube externally densely glandular	Pink to reddish-purple	3, Free	(15.0 -) 17.7 (- 21.0)
***Commicarpus helenae***	(4.5 -) 5.9 (- 10.0)	(1.0 -) 2.0 (- 3.0)	(1.5 -) 2.0 (- 3.0)	(2.0-) 2.5 (- 4.0)	Widely infundibuliform with an extremely short tube	Pink	2, Free	(2.0 -) 2.8 (- 4.0)
***Commicarpus mistus***	(11.0 -) 15.5 (- 21.0)	(5.0 -) 6.5 (- 8.0)	(4.0 -) 5.0 (- 7.0)	(2.0 -) 4.5 (- 6.0)	Narrowly infundibuliform with a distinct puberulent tube	Pink to deep magenta	2-3, Free	(14.0 -) 16.7 (- 20.0)
***Commicarpus plumbagineus***	(12.0 -) 17.0 (- 22.0)	(4.0 -) 4.9 (- 6.0)	(5.0 -) 6.6 (- 9.0)	(3.0 -) 5.2 (- 7.0)	Narrowly infundibuliform with a distinct tube	White	3, Free	(11.0-) 15.2 (- 17.0)
***Commicarpus sinuatus***	(9.0 -) 12.7 (- 16.0)	(1.0 -) 1.6 (- 2.0)	(6.0 -) 7.1 (- 9.0)	(2.0 -) 3.5 (- 5.0)	Narrowly infundibuliform with a distinct tube	Pinkish to purple	3 or 4, Free	(15.0 -) 18.1 (- 22.0)

**Fig 7:**
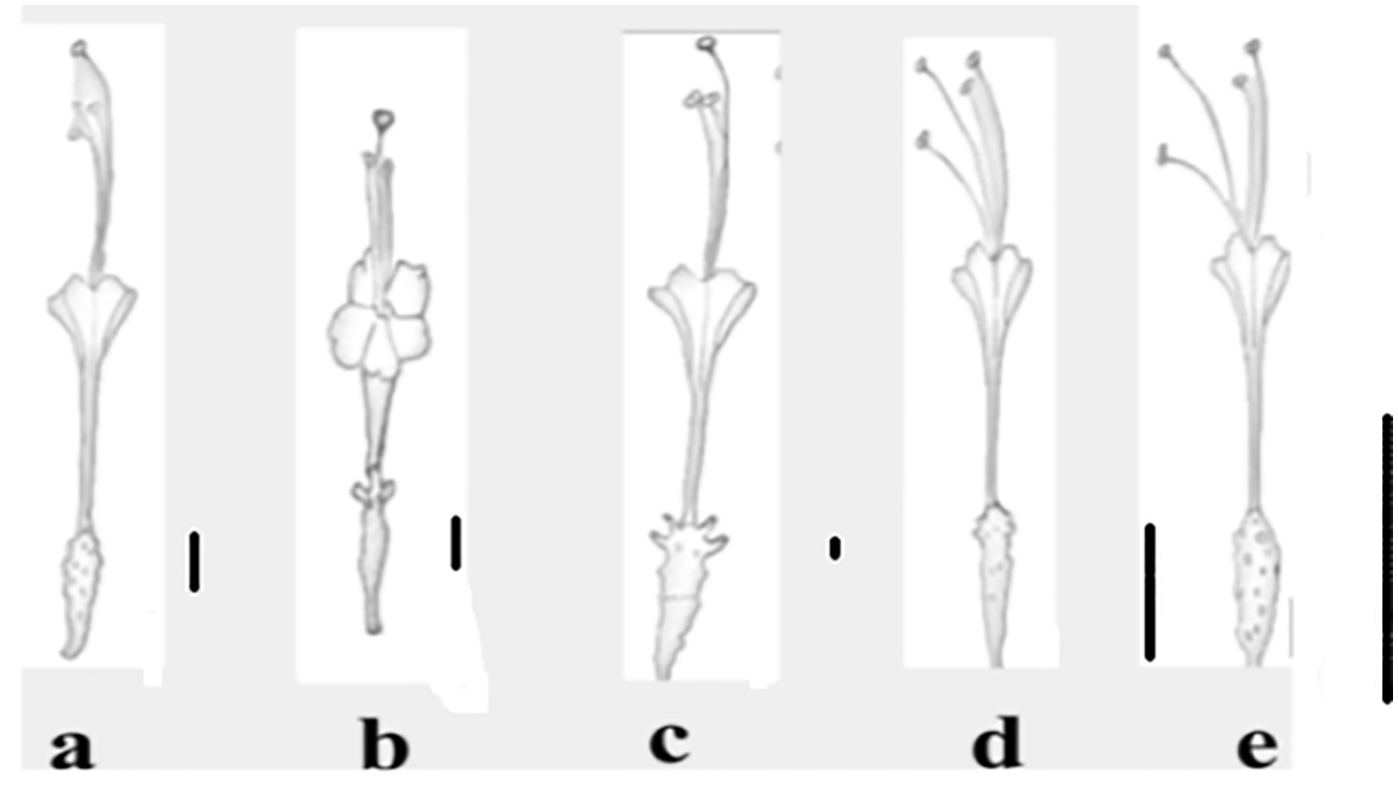
Flowers of *Commicarpus* species: (a) *C. grandiflorus*; (b) *C. helenae*; (c) *C. mistus*; (d) *C. plumbagineus*; (e) *C. sinuatus.* Scale bars 10 mm.

Flower size varies considerably among species, with *C. helenae* possessing the smallest flowers, measuring (4.5–) 5.9 (–10.0) mm, and *C. plumbagineus* exhibiting the largest, ranging from (12.0–) 17 (–22.0) mm. Pedicel length also varies, with *C. sinuatus* having the shortest pedicels (1.0–) 1.6 (–2.0) mm, while *C. mistus* possesses the longest (5.0–) 6.5 (–8.0) mm.

#### Petaloid region and coriaceous segment lengths (Fig 7 and Table 5).

The length of petaloid region segments differs among species. *C. grandiflorus* and *C. sinuatus* exhibit the longest petaloid region segments, measuring (6.0–) 7.5 (–9.0) mm and (6.0–) 7.1 (–9.0) mm respectively. In contrast, *C. helenae* has the shortest petaloid region segments, measuring (1.5–) 2.0 (–3.0) mm. Coriaceous segment length is greatest in *C. plumbagineus* (3.0–) 5.2 (–7.0) mm and shortest in *C. helenae* (2.0–) 2.5 (–4.0) mm.

#### Perigonium morphology and colour (Fig 7 and Table 5).

Perigonium morphology presents notable interspecific variation. Four species (*C. grandiflorus*, *C. mistus*, *C. plumbagineus*, and *C. sinuatus*) share a narrowly infundibuliform perigonium shape, though distinctions exist in the tube characteristics. *C. grandiflorus* features a distinct tube densely covered with external glandular hairs. *C. plumbagineus* has a distinct tube, while *C. mistus* displays a puberulent tube. In contrast, *C. helenae* possesses a widely infundibuliform perigonium with an extremely short tube, setting it apart from the others. Perigonium colour varies among species. *C. grandiflorus* exhibits pink to reddish-purple perigonia, while *C. helenae* presents pink flowers. *C. mistus* has pink to deep magenta perigonia, *C. plumbagineus* is distinguished by its pure white flowers, and *C. sinuatus* displays pinkish to purple perigonia.

#### Stamen characteristics (Fig 7 and Table 5).

The number of stamens differs slightly among species. *C. grandiflorus* and *C. plumbagineus* each have three stamens, whereas *C. mistus* possesses two to three stamens. *C. sinuatus* has three to four stamens, while *C. helenae* typically has two stamens.

Stamen length also varies considerably. *C. helenae* exhibits the shortest stamens, measuring (2.0–) 2.8 (–4.0) mm, whereas *C. sinuatus* has some of the longest stamens, reaching (15–) 18.1 (–22) mm.

#### Ovary and stigma structure (Figs 5 and 6 and Table 4).

All *Commicarpus* species examined in this study produce bisexual flowers with ellipsoid ovaries, each containing a single ovule. The stigma is consistently exerted and capitate across all species.

#### Fruit morphology (Fig 8 and Table 4).

The fruits of the *Commicarpus* genus exhibit variations in shape, length, and indumentum across species. *C. grandiflorus*, *C. helenae*, *C. mistus*, and *C. sinuatus* produce clavate fruits, whereas *C. plumbagineus* has fusiform-shaped fruits.

**Fig 8:**
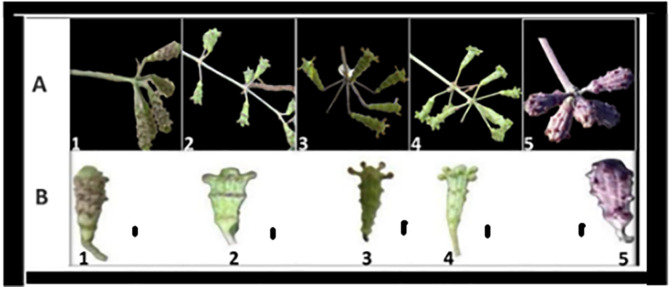
Illustrations of *Commicarpus* species: (A) fruiting stems, (B) fruit photo, ((1) *C. grandiflorus*; (2) *C. helenae*; (3) *C. mistus*; (4) *C. plumbagineus*; (5) *C. sinuatus.* Scale bars 1 mm.

Fruit length varies among species. *C. grandiflorus* produces fruits measuring (5.0–) 6.3 (–7.0) mm, while *C. helenae* has slightly smaller fruits ranging from (4.0–) 4.8 (–6.0) mm. The longest fruits are found in *C. plumbagineus*, measuring (7.0–) 8.0 (–9.0) mm, followed by *C. mistus* at (6.0–) 7.4 (–8.0) mm and *C. sinuatus* at (5.0–) 6.7 (–8.0) mm.

The indumentum (fruit surface covering) is highly species-specific. *C. grandiflorus* fruits are covered with numerous sessile glands and finely viscid pubescence, contributing to a sticky texture. *C. helenae* fruits have long-stalked glands at the apex, with inconspicuous glandular features on the lower parts. *C. mistus* exhibits puberulent hairs along with prominent long-stalked glands at the apex. In contrast, *C. plumbagineus* fruits are covered with prominent sessile glands, primarily concentrated at the apex, while *C. sinuatus* fruits feature sessile glands scattered over the entire surface. Bracts are present in *C. helenae* but absent in the other species.

### Anatomical analysis

#### Stem anatomy (Fig 9 and Table 6).

The anatomical characteristics of the stem in five *Commicarpus* species were analyzed based on their transverse sections. The results reveal variations in the structure of the cortex, vascular cylinder, and parenchyma layers across the studied taxa, as summarized below.

**Table 6:** A summary of the anatomical characteristics observed in transverse sections of the stem of *Commicarpus* species in Saudi Arabia.

Species	Characters					
	Stem					
	Cortex			Vascular cylinder		No. of parenchyma layers between outer and inner vascular
	No. of the cortex layers					
	Angular collenchyma	Chlorenchyma	Endodermis	Inner bundles		
				Large	Small	
***Commicarpus grandiflorus***	1 - 2	5 - 6	1	2	4	3–4
***Commicarpus helenae***	2 – 3	3 - 4	1	2	6	4–8
***Commicarpus mistus***	1 – 2	3 - 4	1	3	6	3–4
***Commicarpus plumbagineus***	1 – 2	3 - 4	1	2	6	4–5
***Commicarpus sinuatus***	2 – 3	2 - 3	1	2	6	4–7

**Fig 9:**
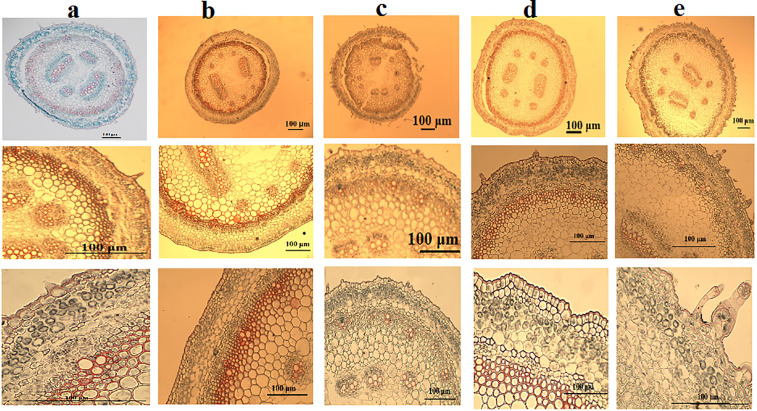
Stem anatomy of *Commicarpus* species studied (cross sections): (a) *C. grandiflorus*; (b) *C. helenae*; (c) *C. mistus*; (d) *C. plumbagineus*; (e) *C. sinuatus.* Scale bar is 100 μm.

#### Cortex structure.

The cortex of the examined species consists of collenchyma, chlorenchyma, and endodermis. The number of cortex layers varies significantly between species:

The cortical region shows variation across species in both angular collenchyma and chlorenchyma layers. Angular collenchyma range from 1–2 layers in *C. grandiflorus*, *C. mistus*, and *C. plumbagineus*, while *C. helenae* and *C. sinuatus* possess 2–3 layers. The chlorenchyma layer configuration varies more substantially, with *C. grandiflorus* showing the highest number (5–6 layers), while other species typically display 3–4 layers, except for *C. sinuatus* which has 2–3 layers. All species share a consistent single compact, barrel-shaped parenchymatous cells endodermis layer.

#### Vascular cylinder.

Stems are in secondary growth in the outer region. In this way, there are no outer vascular bundles, as these bundles are typical of primary growth. The outer vascular cylinder is continuous, consisting of secondary xylem and phloem in all species. This secondary xylem is composed of numerous libriform fibers interspersed with groups of xylem vessels. The medullary vascular bundles, however, are in primary growth, as they do not form continuous cylinders.

The inner vascular region shows two distinct bundle types:

- Large bundles: Most species contain 2 large bundles, with *C. mistus* showing slight variation 3 large bundles.
- Small bundles: *C. grandiflorus* contains 4 small bundles, while all other species possess 6 small bundles.

#### Parenchyma layers.

The number of parenchyma layers between outer and inner vascular tissues shows considerable variation among species:

- *C. helenae* exhibits the widest range (4–8 layers).
- *C. sinuatus* shows moderate variation (4–7 layers)
- *C. plumbagineus* contains 4–5 layers.
- *C. grandiflorus* and *C. mistus* display the narrowest range (3–4 layers).

#### Leaf anatomy (Fig 10 and Table 7).

The anatomical characteristics of the leaf structure in five *Commicarpus* species were analyzed using transverse sections, with observations focusing on epidermal thickness, palisade and spongy tissue thickness, and midrib structure. The findings are summarized below.

**Table 7:** A summary of the anatomical characteristics observed in transverse sections of the leaf of *Commicarpus* species in Saudi Arabia.

Species	Characters					
	Leaf					
	Epidermal		Palisade thickness (µm)	Spongy thickness (µm)	Midrib	
	Thickness (µm)					
	Adaxial	Abaxial			Adaxial	Abaxial
*Commicarpus grandiflorus*	(8.58 -) 9.47 (- 11.29)	(5.26 -) 6.09 (- 7.27)	(84.49 -) 93.43 (- 99.47)	(74.24 -) 79.47 (- 84.79)	Flat	Concave
*Commicarpus helenae*	(9.75 -) 15.55 (- 19.79)	(5.71 -) 7.57 (- 9.65)	(78.04 -) 83.38 (- 88.08)	(44.09 -) 54.92 (- 67.70)	Slightly concave	Concave
*Commicarpus mistus*	(11.50 -) 16.51 (- 20.57)	(10.29 -) 12.85 (- 16.59)	(38.92 -) 48.46 (- 54.76)	(55.52 -) 65.78 (-71.74)	Slightly convex	Flat
*Commicarpus plumbagineus*	(9.98 -) 13.55 (- 17.74)	(8.32 -) 12.32 (- 15.12)	(50.33 -) 65.27 (- 84)	(56.32 -) 58.36 (- 61.49)	Flat	Flat
*Commicarpus sinuatus*	(8.29 -) 8.89 (- 9.57)	(6.38 -) 8.03 (- 8.99)	(30.57 -) 36.01 (- 41.28)	(54.22 -) 61.61 (- 72.26)	Slightly convex	Flat

**Fig 10:**
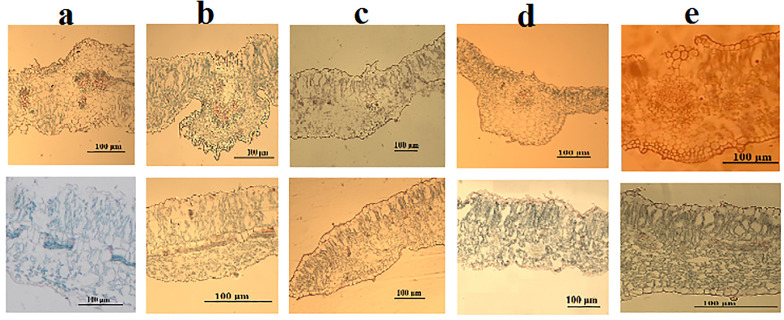
Leaf anatomy of *Commicarpus* species studied (cross sections): (a) *C. grandiflorus*; (b) *C. helenae*; (c) *C. mistus*; (d) *C. plumbagineus*; (e) *C. sinuatus.* Scale bar is 100 μm.

#### Epidermal thickness.

The adaxial and abaxial epidermal thickness varies considerably among the studied species. *C. mistus* exhibits the thickest adaxial epidermis (11.50–20.57 µm), followed by *C. helenae* (9.75–19.79 µm) and *C. plumbagineus* (9.98–17.74 µm). In contrast, *C. sinuatus* and *C. grandiflorus* show thinner adaxial epidermal layers, ranging from 8.29–9.57 µm and 8.58–11.29 µm, respectively.

The abaxial epidermal thickness follows a similar trend, with *C. mistus* presenting the thickest layer (10.29–16.59 µm), followed by *C. plumbagineus* (8.32–15.12 µm) and *C. helenae* (5.71–9.65 µm). *C. grandiflorus* and *C. sinuatus* again display thinner abaxial layers, ranging from 5.26–7.27 µm and 6.38–8.99 µm, respectively.

#### Palisade and spongy mesophyll thickness.

Palisade mesophyll thickness shows notable interspecific variation. *C. grandiflorus* exhibits the thickest palisade layer (84.49–99.47 µm), followed by *C. helenae* (78.04–88.08 µm). In contrast, *C. sinuatus* has the thinnest palisade mesophyll (30.57–41.28 µm), indicating reduced photosynthetic tissue compared to other species. Intermediate values are observed in *C. plumbagineus* (50.33–84.00 µm) and *C. mistus* (38.92–54.76 µm).

The spongy mesophyll thickness also varies, with *C. grandiflorus* showing the greatest spongy layer thickness (74.24–84.79 µm). *C. sinuatus* and *C. mistus* exhibit moderately thick spongy mesophylls (54.22–72.26 µm and 55.52–71.74 µm, respectively), while *C. plumbagineus* (56.32–61.49 µm) and *C. helenae* (44.09–67.70 µm) show slightly thinner layers.

#### Midrib structure.

The structural configuration of the midrib displays significant variation among the species. The adaxial surface of the midrib in *C. grandiflorus* and *C. plumbagineus* is flat, while *C. helenae* shows a slightly concave adaxial surface, and *C. mistus* and *C. sinuatus* exhibit slightly convex adaxial surfaces. The abaxial surface varies from convex in *C. grandiflorus* and *C. helenae* to flat in *C. mistus*, *C. plumbagineus*, and *C. sinuatus*.

#### Petiole anatomy (Fig 11 and Table 8).

The petiole anatomical characteristics of five *Commicarpus* species were analyzed based on transverse sections, focusing on petiole shape, ground tissue structure, and vascular tissue. The results demonstrate significant interspecific variation as outlined below.

**Table 8:** A summary of the anatomical characteristics observed in transverse sections of the petiole of *Commicarpus* species in Saudi Arabia.

Species	Characters										
	Petiole										
	Petiole shape	Petiole outline		Ground tissue			Vascular tissue				
		Adaxial	Abaxial	Collenchyma layers	Parenchyma layers						
					Over bundles	Lower bundles	Main bundles No.	The presence of interspersed bundles	Number of interspersed bundles	Main bundles arranged on	Lateral bundles No.
***Commicarpus grandiflorus***	Cup-shaped	Deep concave	Convex	01-Mar	03-Apr	05-Jun	3	-	0	Open arc	2
***Commicarpus helenae***	Cup-shaped	Deep concave	Convex	02-May	03-Jun	04-May	4	+	1	Deep arc	2
***Commicarpus mistus***	Arc-shaped	Deep concave	Flat	01-Feb	02-Apr	04-May	3	-	0	Open arc	2
***Commicarpus plumbagineus***	Cup-shaped	Very deep concave	Flat	02-Mar	05-Aug	06-Jul	5	+	2	Deep arc	3 (2 + 1)
***Commicarpus sinuatus***	Arc-shaped	Semi-concave	Flat	02-Mar	03-May	05-Jun	3	+	2	Open arc	2

**Fig 11:**
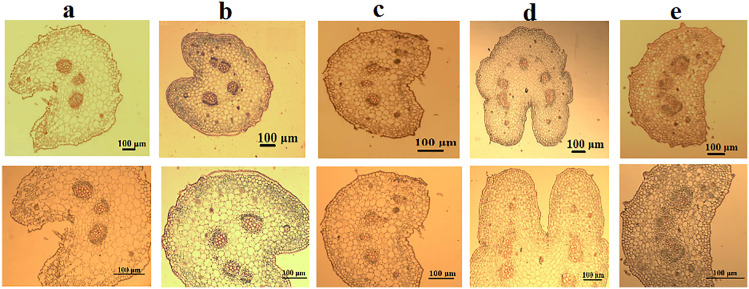
Petiole anatomy of *Commicarpus* species studied (cross sections): (a) *C. grandiflorus*; (b) *C. helenae*; (c) *C. mistus*; (d) *C. plumbagineus*; (e) *C. sinuatus.* Scale bar is 100 μm.

#### Petiolar outline and shape.

The petiole outline exhibits notable interspecific variation, particularly in shape and concavity. *C. grandiflorus*, *C. helenae*, and *C. plumbagineus* have cup-shaped petioles, with *C. plumbagineus* displaying the deepest concavity (very deep concave), while *C. grandiflorus* and *C. helenae* are characterized by deep concavity. In contrast, *C. mistus* and *C. sinuatus* exhibit arc-shaped petioles, with the adaxial surface being deep concave in *C. mistus* and slightly concave in *C. sinuatus*. The abaxial surface across all species remains consistently concave.

#### Ground tissue structure.

The ground tissue structure, comprising collenchyma and parenchyma layers, varies among species. Collenchyma layers, located beneath the epidermis, range from 1–3 layers in *C. grandiflorus* to 2–3 layers in *C. plumbagineus*, and *C. sinuatus*, while *C.mistus* from 1–2–2–5 in *C. helenae*.

Parenchyma layers are distributed over and below the vascular bundles. *C. plumbagineus* shows the most extensive parenchyma development, with 5–8 layers over the bundles and 6–7 layers below. *C. grandiflorus* and *C. sinuatus* have 3–4 and 3–5 layers over the bundles, respectively, with 5–6 layers below. *C. helenae* presents moderate development with 3–6 layers over and 4–5 layers below the bundles, while *C. mistus* has the least extensive parenchyma (2–4 layers over and 4–5 layers below).

#### Vascular tissue organization.

The vascular tissue displays considerable variation in the number, arrangement, and distribution of vascular bundles. The number of main vascular bundles ranges from three in *C. grandiflorus*, *C. mistus*, and *C. sinuatus* to four in *C. helenae* and five in *C. plumbagineus*.

The arrangement of the main bundles is species-specific. *C. grandiflorus*, *C. mistus*, and *C. sinuatus* display an open arc formation, while *C. helenae* and *C. plumbagineus* show a deep arc configuration.

Small vascular bundles are present in *C. helenae*, *C. plumbagineus*, and *C. sinuatus* but absent in *C. grandiflorus* and *C. mistus*. *C. helenae* has one small bundle, while *C. plumbagineus* and *C. sinuatus* possess two each.

#### Lateral bundles.

The number of lateral vascular bundles also varies. Most species, including *C. grandiflorus*, *C. helenae*, *C. mistus*, and *C. sinuatus*, have two lateral bundles. In contrast, *C. plumbagineus* features three lateral bundles, arranged as two primary bundles with an additional smaller bundle (2 + 1), which may further enhance vascular efficiency.

### Palynological analysis (Figs 12 and 13 and Table 9)

According to Erdtman [28]; if P/E is 88–100 or 100–114, so pollen grains will be oblate-spheroidal or prolate-spheroidal shapes respectively as *Commicarpus* species exhibited knowing that 100 refers to apolar pollen grains. *Commicarpus* pollen grains were classified as large to very large. They were pantoporate, with tubuliferous and spinulose tectum.

**Table 9:** A summary of the morphological characteristics of *Commicarpus* species in Saudi Arabia.

Species	Polar axis (µm)	Equatorial axis (µm)	P/E x 100 (µm)	Shape class	Size of pollen grains	Sculpturing	Tubuliferous Density	Length of spinules (µm)	Diameter of pores (µm)
***Commicarpus grandiflorus***	(98.09 -) 108.04 (- 119.46)	(99.70 -) 114.06 (- 128.55)	(92.93 -) 94.7 (- 98.38)	Oblate spheroidal	Very large grains	Tubuliferous and Spinulose	High density	(1.21 -) 2.21 (- 3.17)	(4.19 -) 4.52 (- 4.87)
***Commicarpus helenae***	(58.91-) 62.03 (- 65.40)	(58.56 -) 60.41 (- 62.37)	(100.59 -) 102.68 (- 104.86)	Prolate spheroidal	Large grains	Tubuliferous and Spinulose	Low density	(1.63 -) 2.02 (- 2.79)	(1.60 -) 2.03 (- 2.63)
***Commicarpus mistus***	(91.87 -) 93.58 (- 95.49)	(88.53 -) 90.40 (- 92.46)	(103.27 -) 103.53 (- 103.77)	Prolate spheroidal	Large grains	Tubuliferous and Spinulose	High density	(1.37 -) 1.95 (- 2.39)	(1.91 -) 2.23 (- 2.27)
***Commicarpus plumbagineus***	(106.38 -) 117.02 (- 127.75)	(101.28 -) 111.50 (-122.65)	(104.16 -) 104.95 (- 105.05)	Prolate spheroidal	Very large grains	Tubuliferous and Spinulose	High density	(1.16 -) 1.82 (- 2.52)	(3.23 -) 3.97 (- 4.59)
***Commicarpus sinuatus***	(110.52 -) 111.14 (- 111.81)	(103.02 -) 106.35 (-110.05)	(101.59 -) 104.51 (-107.28)	Prolate spheroidal	Very large grains	Tubuliferous and Spinulose	Low density	(1.25 -) 1.59 (- 2.22)	(2.35 -) 3.69 (- 4.84)

**Fig 12:**
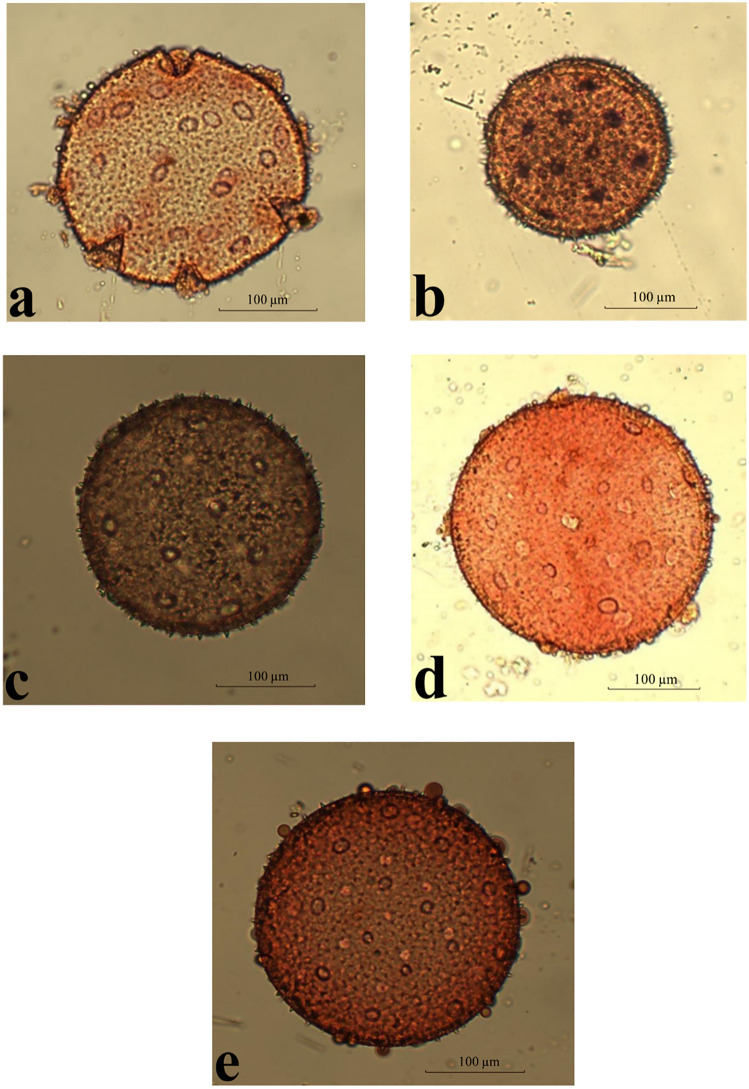
Pollen grains under polar view for *Commicarpus* species studied (light microscope): (A) *C. grandiflorus*; (B) *C. helenae*; (C) *C. mistus*; (D) *C. plumbagineus*; (E) *C. sinuatus.*

**Fig 13:**
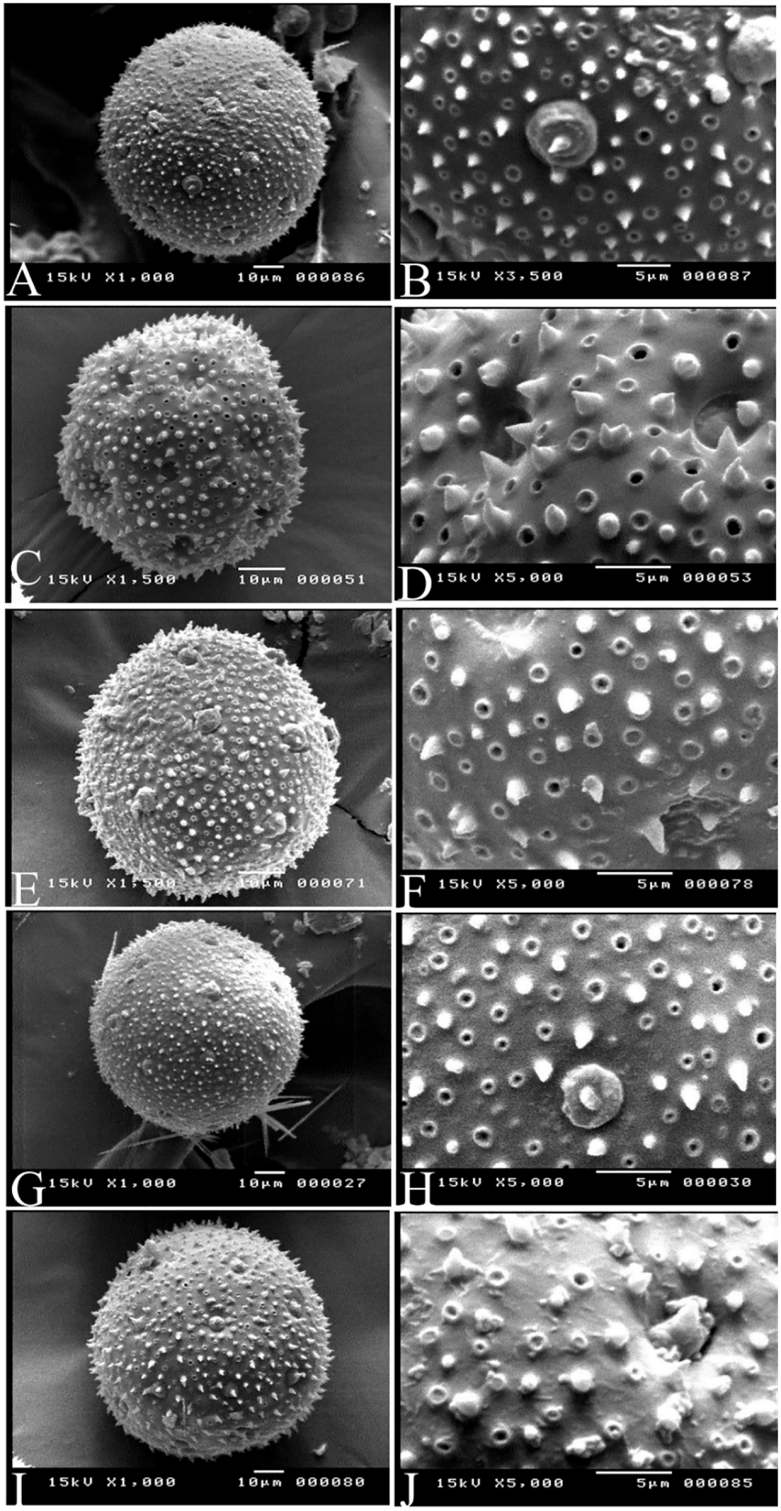
Pollen grains under polar view for *Commicarpus* species studied (scanning electron microscope): (A, B) *C. grandiflorus*; (C, D) *C. helenae*; (E, F) *C. mistus*; (G, H) *C. plumbagineus*; (I, J) *C. sinuatus.*

Morphometric analysis revealed significant interspecific variation in polar and equatorial axes. *C. plumbagineus* had the largest polar axis, measuring (106.38–) 117.06 (–127.75) μm, while *C. grandiflorus* exhibited the largest equatorial axis, ranging from (99.70–) 114.02 (–128.55) μm. In contrast, *C. helenae* possessed the smallest pollen grains, with polar and equatorial axis measurements of (58.91–) 62.03 (–65.40) μm and (58.56–) 60.41 (–62.37) μm, respectively.

Pollen grain size classification varies among species. *C. helenae* and *C. mistus* had large grains. Conversely, *C. grandiflorus*, *C. plumbagineus* and *C. sinuatus* were characterized by very large grains. Shape also served as a distinguishing feature, with *C. grandiflorus* pollen grains classified as oblate-spheroidal, whereas the remaining four species (*C. helenae*, *C. mistus*, *C. plumbagineus*, and *C. sinuatus*) displayed a prolate-spheroidal form.

The P/E ratio further supported these findings. *C. grandiflorus* exhibited a P/E ratio of (92.93–) 94.07 (–98.38) μm, which is below the 100 μm threshold typically used to differentiate between oblate and prolate spheroidal shapes. In contrast, the remaining species had P/E ratios exceeding 100 μm, confirming their prolate-spheroidal classification.

Tubuliferous density and pore size also varies among species. *C. grandiflorus*, *C. mistus*, and *C. plumbagineus* exhibited higher tubuliferous densities compared to *C. helenae* and *C. sinuatus*. The largest pore diameter was recorded in *C. grandiflorus* (4.19–) 4.52 (–4.87) μm, while the smallest was observed in *C. mistus* (1.91–) 2.23 (–2.27) μm and *C. helenae* (1.60–) 2.03 (–2.63) μm.

Spinule length also varies among species. *C. grandiflorus* exhibited the longest spinules, ranging from (1.21–) 2.21 (–3.17) μm, whereas *C. sinuatus* had the shortest (1.25–) 1.59 (–2.22) μm.

### Scoring data and statistical analysis

**Fig 14:**
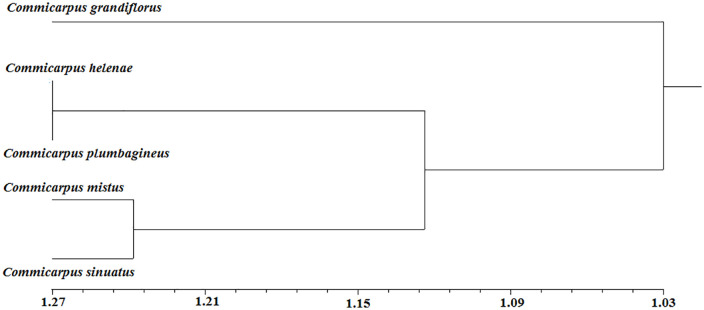
Phenogram of *Commicarpus* species studied.

The collective taxonomic traits were scored to be 54 parameters distinguished as 31 morphological, 15 anatomical and 8 palynological traits (Tables 10 and 11) among *Commicarpus* species. The operational taxonomic units (OTUs) of all studied species are present at 1.03 where *C. grandiflorus* is split as delimited genera. At 1.123 OTUs, there are two groups; the first group contains *C. helenae* and *C. plumbagineus* while the second group contains *C. mistus* and *C. sinatus* at 1.24 OTUs (Fig 14). According to similarity matrix (Table 12), *C. grandiflorus* and *C. helenae* are regarded as the distant plant species while *C. helenae* and *C. plumbagineus, C. helenae* and *C. sinuatus* besides *C. mistus* and *C. sinuatus* are the close related plant species.

**Table 10:** Characters and character states scored in the numerical analysis.

	Character	Character States	Type of Character
1	Habit	Perennial herbs, soft woody = 0	Binary
		Perennial herbs, woody-based = 1	
2	Branching pattern	Slightly branched = 0	Binary
		Much branched = 1	
3	Growth orientation	accumbent/ascending = 0	Multi
		Suberect to Scrambling = 1	
		Erect = 2	
		Scandent or Trailing = 3	
		Tangled = 4	
4	Stem length (Mean)	m	Continuous
5	Stem hairs	Glabrous = 0	Multi
		Puberulent =1	
		Pilose-glandular hairs = 2	
6	Stem texture	Non-sticky = 0	Binary
		Sticky = 1	
7	Leaf length (Mean)	mm	Continuous
8	Petiole length (Mean)	mm	Continuous
9	Leaf texture	Non-fleshy = 0	Binary
		Fleshy = 1	
10	Leaf shape	Ovate-triangular = 0	Multi
		Broadly ovate = 1	
		Ovate or suborbicular = 2	
		Sinuate or lobed = 3	
11	Lamina length (Mean)	mm	Continuous
12	Lamina width (Mean)	mm	Continuous
13	Lamina apex	Acute or obtuse = 0	Multi
		Obtuse or rounded = 1	
		Obtuse = 2	
14	Lamina base	Truncate to subcordate = 0	Multi
		Truncate to broadly cuneate = 1	
		Cordate = 2	
15	Lamina margin	Entire = 0	Multi
		Entire or sinuately lobed = 1	
		Entire or obscurely sinuate = 2	
		Entire to irregularly sinuate = 3	
		Sinuately lobed = 4	
16	Flower length (Mean)	mm	Continuous
17	Peduncle length (Mean)	mm	Continuous
18	Petaloid region length (Mean)	mm	Continuous
19	Coriaceous length (Mean)	mm	Continuous
20	Perigonium shape	Narrowly infundibuliform, with a distinct tube externally densely glandular = 0	Multi
		Narrowly infundibuliform with a distinct puberulent tube = 1	
		Narrowly infundibuliform with a distinct tube = 2	
		Widely infundibuliform with an extremely short tube = 3	
21	Perigonium color	Pink to reddish-purple = 0	Multi
		Pink = 1	
		Pink to deep magenta = 2	
		White = 3	
		Pinkish to purple = 4	
22	Stamen number	2-3 = 0	Binary
		3 or 4 = 1	
23	Stamen length	mm	Continuous
24	Inflorescence type	Umbels = 0	Binary
		Whorls = 1	
25	Inflorescence hair	Glabrous = 0	Continuous
		Puberulent =1	
		Glandular – hairy = 2	
26	Inflorescence texture	Non-sticky = 0	Binary
		Sticky = 1	
27	Inflorescence length (Mean)	mm	Continuous
28	Fruit shape	Clavate = 0	Binary
		Fusiform = 1	
29	Fruit length (Mean)	mm	Continuous
30	Fruit indumentum	Fruit covered with numerous sessile glands and finely viscid pubescent = 0	Multi
		Fruit covered with long-stalked glands at the tip, inconspicuous glandular below = 1	
		Fruit covered with puberulent hairs and prominent long-stalked glands at the tip, inconspicuous glandular below = 2	
		Fruit covered with prominent sessile glands, mostly at the apex = 3	
		Fruit covered with sessile glands, scattered all over = 4	
31	Bracts	Absent = 0	Binary
		Present = 1	
32	Number of large inner bundles in stem	2 = 0	Binary
		3 = 1	
33	Number of small inner bundles in stem	4 = 0	Binary
		6 = 1	
34	Thickness of the adaxial epidermal cell in transverse section of the leaf (Mean)	µm	Continuous
35	Thickness of the abaxial epidermal cell in transverse section of the leaf (Mean)	µm	Continuous
36	Thickness of the palisade of leaf (Mean)	µm	Continuous
37	Thickness of the spongy leaf (Mean)	µm	Continuous
38	Leaf midrib shape at adaxial	Flat = 0	Multi
		Slightly concave = 1	
		Slightly convex = 2	
39	Leaf midrib shape at abaxial	Flat = 0	Binary
		Concave = 1	
40	Petiole outline shape in transverse section	Cup-shaped = 0	Binary
		Arc-shaped = 1	
41	Petiole’s adaxial surface	Deep concave = 0	Multi
		Very deep concave = 1	
		Slightly concave = 2	
42	Number of the petiole main bundles	3 = 0	Multi
		4 = 1	
		5 = 2	
43	The presence of interspersed bundles	Without interspersed bundles = 0	Binary
		With interspersed bundles = 1	
44	Number of the interspersed bundles	3 = 0	Multi
		4 = 1	
		5 = 2	
45	Main bundles shape	Open arc = 0	Binary
		Deep arc = 1	
46	Number of the lateral bundles	2 = 0	Binary
		3 = 1	
47	Polar pollen length (Mean)	µm	Continuous
48	Equatorial pollen length (Mean).	µm	Continuous
49	P/E ratio (Mean)	µm	Continuous
50	Pollen shape	Oblate spheroidal = 0	Binary
		Prolate spheroidal = 1	
51	Pollen size	Large = 0	Binary
		Very large = 1	
52	Tubuliferous density	Low density = 0	Binary
		High density = 1	
53	Length of spinules (Mean)	µm	Continuous
54	Diameter of pores (Mean)	µm	Continuous

**Table 11:** Data matrix for the Numerical Taxonomy (the characters used are morphological, anatomical and palynological).

Taxa	Characters																	
	1	2	3	4	5	6	7	8	9	10	11	12	13	14	15	16	17	18
***Commicarpus grandiflorus***	0	0	0	1.8	2	1	33.3	8.1	0	0	25.6	17.9	0	0	0	16.6	6.2	7.5
***Commicarpus helenae***	1	1	1	1.3	0	0	24.1	5.9	1	1	17.8	14.8	0	2	1	5.9	2	2
***Commicarpus mistus***	1	0	2	0.8	1	0	13.7	3.5	1	2	12.2	7.3	1	1	2	15.5	6.5	5
***Commicarpus plumbagineus***	0	0	3	1.9	0	0	38.5	11.6	0	1	29.5	24.4	0	1	3	17	4.9	6.6
***Commicarpus sinuatus***	1	1	4	2.4	0	0	10.5	2	1	3	8.7	7.7	2	0	4	12.7	1.6	7.1
**Taxa**	**Characters**																	
	19	20	21	22	23	24	25	26	27	28	29	30	31	32	33	34	35	36
***Commicarpus grandiflorus***	3.3	0	0	0	17.7	0	2	1	34.8	0	6.3	0	0	0	0	9.47	6.09	93.43
***Commicarpus helenae***	2.5	3	1	0	2.8	1	0	0	51.2	0	4.8	1	1	0	1	15.55	7.57	83.38
***Commicarpus mistus***	4.5	1	2	0	16.7	0	1	0	24.2	0	7.4	2	0	1	1	16.51	12.85	48.46
***Commicarpus plumbagineus***	5.2	2	3	0	15.2	1	0	0	42.5	1	8	3	0	0	1	13.55	12.32	65.27
***Commicarpus sinuatus***	3.5	2	4	1	18.1	0	0	0	22.6	0	6.7	4	0	0	1	8.89	8.03	36.01
**Taxa**	**Characters**																	
	37	38	39	40	41	42	43	44	45	46	47	48	49	50	51	52	53	54
***Commicarpus grandiflorus***	79.47	0	1	0	0	0	0	0	0	0	108.04	114.06	94.7	0	1	1	2.21	4.52
***Commicarpus helenae***	54.92	1	1	0	0	1	1	1	1	0	62.03	60.41	102.68	1	0	0	2.02	2.03
***Commicarpus mistus***	65.78	2	0	1	0	0	0	0	0	0	93.58	90.4	103.53	1	0	1	1.95	2.23
***Commicarpus plumbagineus***	58.36	0	0	0	1	2	1	2	1	1	117.02	111.5	104.95	1	1	1	1.82	3.97
***Commicarpus sinuatus***	61.61	2	0	1	2	0	1	2	0	0	111.14	106.35	104.51	1	1	0	1.59	3.69

**Table 12:** Similarity matrix of studied *Commicarpus* species.

	*C. grandiflorus*	*C. helenae*	*C. mistus*	*C. plumbagineus*	*C. sinuatus*
***C. grandiflorus***	1.00	0.54	0.65	0.59	0.56
***C. helenae***	0.54	1.00	0.74	0.80	0.80
***C. mistus***	0.65	0.74	1.00	0.68	0.80
***C. plumbagineus***	0.59	0.80	0.68	1.00	0.74
***C. sinuatus***	0.56	0.80	0.80	0.74	1.00

**Fig 15:**
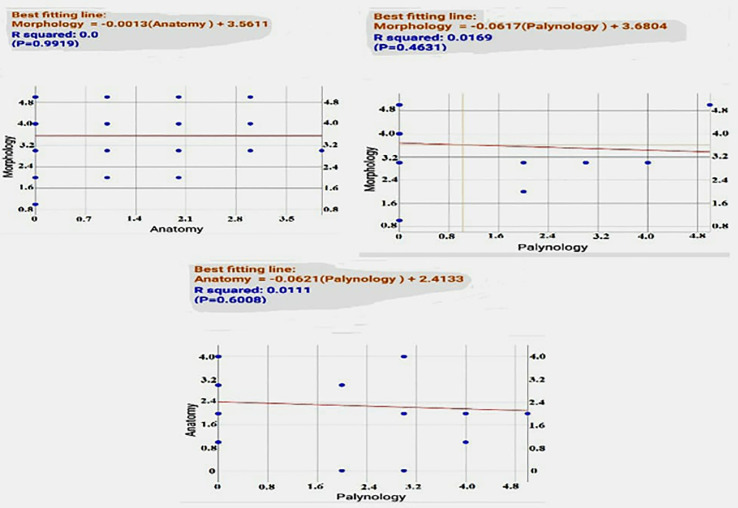
Simple Linear Regression (SLR) of *Commicarpus* species.

Moreover, *P* values of Pearson correlation coefficients determined the highest value between morphological and anatomical parameters on the contrary for both morphological and palynological ones. Simple linear regression (SLR) equations represented in the form of scattered plot graphs denoting that morphological vs anatomical parameters expressed as stationary equivalent regression however, other regressed parameters showed the low values (Table 13 and Fig 15).

**Table 13:** *P* values of Pearson correlation coefficients among characteristic parameters.

	Morphology	Anatomy	Palynology
**Morphology**	**–**	**0.99**	**0.46**
**Anatomy**	**0.99**	**–**	**0.60**
**Palynology**	**0.46**	**0.60**	**–**

## Species synopsis

### Taxonomic treatment

*Key of the Commicarpus species in Saudi Arabia based on morphological characteristics.*

1. Stem and inflorescence are sticky, hairy with glandular hairs ……………….*C. grandiflorus*
2. + Stem and inflorescence are non-sticky, glabrous, puberulent hairs …………………………2
3. Leaves are sinuate or lobed; stamen 3 or 4…………………………………...*C. sinuatus*
4. + Leaves are broadly ovate, ovate to sub-orbicular; stamen 2–3………………………………3
5. Leaves broadly ovate, ovate to sub-orbicular; bracts are present; the perigonium is widely infundibuliform with an extremely short tube……………………………………... *C. helenae*
6. + Leaves are broadly ovate, ovate to sub-orbicular; bracts are absent; the perigonium is narrowly infundibuliform with a distinct tube.…………………..............................................4
7. Leaves broadly ovate; perigonium white; fruit fusiform 7–9 mm………...*C. plumbagineus*
8. + Leaves ovate to sub-orbicular; perigonium pink to deep magenta; fruit clavate 6–8 mm……………………………………………………………………………*C. mistus*

*Key of the Commicarpus species in Saudi Arabia based on anatomical characteristics.*

1. Petiole cup-shaped, with adaxial very deep concave, main vascular bundles 5 in deep arc shaped ………………………………………………………………………………….*C.plumbagineus*
2. + Petiole cup- or arc-shaped, with adaxial deep or slightly concave, main vascular bundles 3 or 4 in deep or open arc shaped ………………………………………………………………..2
3. Petiole cup- or arc-shaped, with adaxial deep concave, main vascular bundles 4 in deep arc shaped…………………………………………………………………………. *C.helenae*
4. + Petiole cup- or arc-shaped, with adaxial deep or slightly concave, main vascular bundles 3 in deep or open arc shaped………..............................................................................................3
5. Petiole cup- or arc-shaped, with adaxial slightly concave, main vascular bundles 3 in deep or open arc shaped with two small vascular bundles ………………………………*C.sinuatus*
6. + Petiole cup- or arc-shaped, with adaxial deep concave, main vascular bundles 3 in open arc shaped without small vascular bundles …………………………...…………………………...4
7. Petiole cup-shaped, with adaxial deep concave, main vascular bundles 3 in open arc shaped without small vascular bundles …………………………………………………*C.grandiflorus*
8. Petiole arc-shaped, with adaxial deep concave, main vascular bundles 3 in open arc shaped without small vascular bundles ………………………………………………………*C. mistus*

## Discussion

Morphological traits have historically served as primary taxonomic tools and continue to play a crucial role in contemporary plant systematics. They reveal both external and internal characteristics and are instrumental in interpreting species-level relationships. Morphological variation among *Commicarpus* species likely reflects adaptive responses to arid environments while maintaining taxonomic significance [25].

The present study provides a comprehensive morphological evaluation of five *Commicarpus* species occurring in Saudi Arabia: *C. grandiflorus*, *C. helenae*, *C. mistus*, *C. plumbagineus*, and *C. sinuatus*. The results are compared with the descriptions provided in the foundational works of [11,13], which included all five species, and with [15], whose taxonomic revision was limited to *C. helenae* and *C. plumbagineus*. Overall, the current findings are largely consistent with earlier taxonomic treatments, while offering more precise morphometric data that enhance species delimitation and deepen our understanding of morphological variability.

*Commicarpus grandiflorus* was clearly distinguished by its sticky stems densely covered with pilose-glandular hairs—a defining feature consistently reported by [11,13]. This trait remains taxonomically reliable, as it was not observed in any of the other studied species. The current study also quantified stem length and growth orientation, identifying accumbent to ascending growth forms that had not been previously described in detail.

Leaf morphology exhibited marked interspecific variation, particularly in margin type and texture. *C. grandiflorus* retained its ovate-triangular, entire leaves, as documented in earlier accounts, while *C. helenae* and *C. sinuatus* were confirmed to have sinuate or lobed margins, corroborating the observations of [13]. Quantitative data from the present study—such as petiole length, lamina dimensions, and texture (fleshy vs. non-fleshy)—provided a more rigorous framework for distinguishing among species. The variation in apex morphology among studied species may have ecological implications, influencing water runoff and photosynthetic efficiency [34].

The floral morphology of the examined *Commicarpus* species revealed distinctive features that both confirm and refine previous descriptions [11,13,15]. *C. grandiflorus* and *C. plumbagineus* both exhibited narrowly infundibuliform perigonia with well-developed basal tubes and three free stamens, differing in flower color—pink to reddish-purple in *C. grandiflorus* and white in *C. plumbagineus* [11,13]. *C. helenae* was characterized by a widely infundibuliform perigonium with a short tube, pink coloration, and two stamens, along with sessile and stalked glands on the fruit apex [11,13,15]. *C. mistus* displayed narrowly infundibuliform, deep pink to magenta flowers with a puberulent tube and a variable number of stamens (2–3), while *C. sinuatus* featured pinkish to purple flowers with a prominent floral tube and 3–4 stamens [11,13]. These detailed floral features serve as robust taxonomic indicators for species delimitation within the genus.

Inflorescence and fruit morphology also played a significant role in distinguishing among the species. Inflorescence types were umbellate in *C. grandiflorus*, *C. mistus*, and *C. sinuatus*, and whorled in *C. helenae* and *C. plumbagineus*. Fruit shapes ranged from clavate (*C. grandiflorus*, *C. mistus*, C*. helenae*, *C. sinuatus*) to fusiform (*C. plumbagineus*), in agreement with earlier descriptions and further validated by this study’s observations on gland distribution. Notably, the fruits of *C. grandiflorus* were found to be viscid and densely covered with sessile glands—a feature that aligns closely with the “prominently gland-warted” fruit surfaces described by [11,13].

Although most morphological traits remained stable across geographical distributions, the present study recorded slight variations in some measurements (e.g., leaf and fruit length, perianth size), which may reflect environmental influences or regional morphological plasticity. Observed differences between geographically distinct populations may indicate ecological adaptation and/or underlying genetic divergence. Notably, when comparing *C. helenae* and *C. plumbagineus* populations from Saudi Arabia with their counterparts in southern Africa, as documented by [15], the Saudi specimens exhibited shorter petioles and variations in leaf and floral dimensions, suggesting potential ecological adaptation. These observed differences merit further investigation through molecular analyses to evaluate their phylogenetic implications.

Anatomical characterization serves as a complementary tool to morphological analysis in differentiating species within the same genus. It allows taxonomists to assess how external species appearance correlates with specific habitat types [35]. Anatomical characters, particularly stem and leaf structures, represent reliable systematic markers and are often congruent with molecular phylogenies [36]. The anatomical structures of stems and leaves in *Commicarpus*_species (Nyctaginaceae) align with previous descriptions by [17,18,37]. This study highlights interspecific variations across several anatomical parameters, underscoring adaptations to different environmental conditions and reinforcing their taxonomic significance.

Transverse sections of leaves reveal substantial interspecific differences in epidermal thickness, mesophyll structure, and midrib configuration traits critical for understanding physiological adaptation. The mesophyll in *Commicarpus* species consists of palisade and spongy parenchyma. Palisade cells increase the internal leaf surface, thereby enhancing photosynthesis rates [38], while spongy mesophyll facilitates carbon dioxide circulation throughout the leaf to maintain high photosynthesis rates. Variation in mesophyll organization and vascular bundle architecture suggests functional adaptations related to photosynthetic efficiency and mechanical support. The spongy mesophyll thickness also varies, with *C. grandiflorus* showing the greatest spongy layer thickness (74.24–84.79 µm), suggesting a well-developed internal air space system conducive to efficient gas exchange [39].

*Commicarpus mistus* exhibits the thickest epidermis (adaxial: 11.50–20.57 µm; abaxial: 10.29–16.59 µm). The increased epidermal thickness in *C. mistus* may contribute to enhanced structural support and water retention, reflecting adaptations to drier environments [40]. *C. grandiflorus* presents the thickest palisade (84.49–99.47 µm) and spongy mesophyll layers (74.24–84.79 µm), indicating a high capacity for photosynthesis and gas exchange, consistent with adaptations to arid environments. Conversely, *C. sinuatus* has the thinnest palisade mesophyll (30.57–41.28 µm), potentially reflecting reduced photosynthetic efficiency or adaptation to shaded habitats.

Midrib morphology also varies: very deep concave adaxial surfaces in *C. plumbagineus* likely confer structural rigidity, whereas the semi concave surface observed in *C. sinuatus* may enhance leaf flexibility. These variations in midrib morphology may relate to differences in leaf rigidity and structural support, potentially reflecting species-specific adaptations to varying environmental conditions [41].

The structure of the petiole offers significant taxonomic diagnostic value, as it appears relatively unaffected by environmental variations [37]. Among the studied organs, petiole anatomy provided some of the most taxonomically informative features, particularly in terms of shape, tissue organization, and vascular configuration.

Petiole outlines ranged from cup-shaped in *C. grandiflorus*, *C. helenae*, and *C*. *plumbagineus* to arc-shaped in *C. mistus* and *C. sinuatus*. Ground tissue analysis revealed that *C. plumbagineus* had the most extensive parenchyma development, with up to eight layers above and seven layers below the vascular bundles, suggesting both enhanced mechanical support and storage capacity. In contrast, *C. mistus* had the least developed parenchyma.

The current study reveals notable differences among *Commicarpus* species. *C. grandiflorus* exhibits more rows of chlorenchyma compared to other species. In contrast, *C. helenae* and *C. sinuatus* display a greater number of collenchyma layers (2–3). Collenchyma provides mechanical support to growing parts through thickened walls, which also aids in protection against sunlight and water loss [17,18]. However, the presence of a collenchymatous hypodermis is not considered a xeromorphic adaptation here, as it serves as basic structural support commonly found in many young mesophytic stems.

An examination of the vascular cylinder shows that *C. mistus* possesses three large inner bundles, whereas other species have two. *C. grandiflorus* has four small inner bundles, compared to six in the remaining species. The development of parenchyma between vascular tissues varies among taxa, with *C. helenae* exhibiting the greatest number of layers (4–8), suggesting enhanced storage or internal buffering capacity. In contrast, *C. grandiflorus* and *C. mistus* have fewer layers (3–4), indicating reduced internal differentiation.

Vascular bundle arrangement was species-specific. *C. plumbagineus* exhibited the most complex configuration. This intricate architecture suggests increased hydraulic efficiency and mechanical reinforcement. Small vascular bundles were observed in *C. helenae*, *C. plumbagineus*, and *C. sinuatus*, but were absent in *C. grandiflorus* and *C. mistus*. Their presence may enhance vascular supply and structural flexibility traits associated with evolutionary divergence within Nyctaginaceae. The arrangement of the main bundles among studied species suggests that the differences in vascular support are potential adapted with mechanical stress. Moreover, The presence of the additional bundles (small ones) may contribute to enhanced vascular supply and mechanical stability [42].

A recent study by Pakravan et al. (2023) [19] on the systematics of Nyctaginaceae in Iran, which included *C. helenae*, reveals further differences in these anatomical characteristics. The number of collenchyma layers in the stem and petiole of *C. helenae* observed in the present study is greater than that reported by Pakravan et al. (2023) [19].

The best taxonomic purposes are achieved by combining evidence from different biological fields or levels to illustrate the inter- and intra-relations among different species. The analysis of pollen grain description offers the genetical stable markers for enhancing taxonomic identification [43]. Pollen morphology constitutes a genetically stable and evolutionarily informative dataset, reinforcing its value in taxonomic and phylogenetic inference [44]. The pollen morphology observed in the studied *Commicarpus* species, ranging from prolate spheroidal to oblate spheroidal forms, aligns with characteristics previously reported for other members of the Nyctaginaceae family [6,45].

Although molecular tools have revolutionized plant taxonomy by providing objective, DNA-based evidence to classify plants, resolve complex relationships, and identify cryptic species that are morphologically identical, palynology offers robust morphological markers that assist in the delimitation of closely related species, genera, and families. It is often less affected by ecological conditions compared to genome mutation, offering high genetic stability. Accordingly, palynology plays an incontrovertible role not only in “basic research” concerning botanical taxonomy, phylogeny, phenology and reproductive biology, but also in several fields of applied research that focus on measuring environmental variables focusing on sustainability and climatic changes [46,47].

Struwig et al. (2013) [20] examined the pollen morphology of Southern African *Boerhavia* and *Commicarpus* (Nyctaginaceae). Two *Commicarpus* species in the study which are *C. helenae* and *C. plumbagineus*, both of them occur in Saudi Arabia. The sculpture patterns of these species in the present study are consistent with their findings; however, differences are observed in the length of spinules and pore diameter. Specifically, the spinule length in *C. helenae* is larger than the measurements reported by Struwig et al. (2013) [20]. Conversely, the spinule length in *C. plumbagineus* is smaller than that recorded by Struwig et al. (2013) [20]. Similarly, the pore diameters in *C. helenae* and *C. plumbagineus* are smaller than the values reported by Struwig et al. (2013) [20], respectively.

A more recent study by Pakravan et al. (2023) [19] on the systematics of Nyctaginaceae in Iran, which also included *C. helenae*, reveals further differences in these palynological characteristics. The spinule length of *C. helenae* in this study is smaller than the range reported by Pakravan et al. (2023) [19]. Likewise, the pore diameter of C. *helenae* is also smaller than the range recorded by Pakravan et al. (2023) [19].

Morphologically, *C. grandiflorus* exhibits an oblate spheroidal pollen form with a P/E ratio of [(92.93–) 94.07 (–98.38) μm], which remains below the 100 μm threshold typically used to differentiate between oblate and prolate spheroidal shapes. In contrast, the remaining species in this study have P/E ratios exceeding 100 μm, confirming their classification as prolate spheroidal.

The consistent presence of a tubuliferous and spinulose tectum across all studied species suggests a shared evolutionary trait within the genus. However, the observed variations in polar and equatorial axis dimensions, P/E ratios, spinule length, pore diameter, overall pollen shape, and size provide valuable taxonomic characters for differentiating among these species.

From the scored data and statistical analysis, *C. helenae* and *C. sinuatus* are the transient plant species among studied species, on the other hand, *C. grandiflorus* is the most distant species among them. The morphological and anatomical parameters are the most compatible and homogenetic traits that reflect on each other more than palynological traits. The expression outwards and inwards for plant nature is very susceptible to each other. Hence, adaptable changes within plant species affect the morphological and anatomical nature more than palynological characters. From similarity index, we can observe much more similarity value among *C. helenae*, *C. plumbagineus*, *C. mistus* and *C. sinuatus* with less dissimilarity value between *C.* *grandiflorus* and *C. helenae* only to confirm that all studied species related to each other and not suggest excluding any one as a delimited genera. The congruence among morphological, anatomical, and palynological datasets supports an integrative taxonomic framework for resolving species boundaries within *Commicarpus*. Combing among different plant taxonomic tools including morphology, anatomy and palynology indicate that there is a mutual homogenous compatibility between traditional and modern taxonomic tools to obtain comprehensive optimum clear picture of the taxonomic status for studied species.

The taxonomic keys presented for *Commicarpus* species in Saudi Arabia provide a precise diagnostic framework by combining external morphological traits with internal anatomical features.

The morphological key effectively separates the five species based on observable characters. *C. grandiflorus* is distinct for its sticky, glandular-hairy stems, while *C. sinuatus* is recognized by its sinuate leaves and higher stamen number. The remaining species are differentiated using floral and fruit characteristics, including perigonium shape and color, bract presence, and fruit type (fusiform in *C. plumbagineus* vs. clavate in *C. mistus*). These characters are taxonomically stable and practical for field identification.

The anatomical key adds further resolution, focusing on petiole morphology and vascular bundle configuration. *C. plumbagineus* is unique in having five vascular bundles in a deep arc, while other species show variations in bundle number (three or four), arc shape, and presence or absence of interspersed traces. These internal features are less environmentally influenced, making them valuable for confirming species identity, especially in morphologically similar taxa.

The existence of climate change is confirmed by various evidences from different sources that can be used to reconstruct past climates. Plant species have responded to climate change by range shifting and increasing species richness. Plant taxonomy could be an important way for species distribution to counterbalance rapid climate change. morphological, anatomical and palynological traits are likely to influence the ability of species to take advantage of potentially favorable conditions arising from climate change. Plant species can also adjust to new conditions through phenotypic plasticity [48]. Generally plants respond to climate change impacts in different ways. The dispersal of plant species to new and more favorable sites is the most important plant range shifting in response to climate change impacts. In light of the foregoing, the new *Commicarpus* species are discovered recently at countries close to the same latitude of Jazan region; *C. altus* Thulin from central Somalia and *C. ogadenensis* Thulin from southeastern Ethiopia [49].

## Conclusion

This comprehensive study of *Commicarpus* species in Saudi Arabia has yielded valuable insights into their morphological, anatomical, and palynological diversity. The observed interspecific variations in growth habit, stem and leaf structure, inflorescence and fruit morphology, as well as detailed anatomical and palynological characteristics, have proven to be robust taxonomic markers. The development of identification keys based on both morphological and anatomical features of the petioles enhances the accuracy of species delimitation within the genus in Saudi Arabia. These findings not only facilitate the identification and classification of *Commicarpus* species in the region but also contribute to a deeper understanding of their evolutionary relationships and ecological adaptations. Taxonomy of *Commicarpus* species can estimate the impact of climatic changes on a specific area by classifying, mapping, and monitoring to detect shifts in their distribution, phenology, and community structure. By utilizing historical records and modern biodiversity surveys, taxonomists can measure how species compositions change over time and predict future responses to climate stressors.
